# A Novel Nanosafety Approach Using Cell Painting, Metabolomics, and Lipidomics Captures the Cellular and Molecular Phenotypes Induced by the Unintentionally Formed Metal-Based (Nano)Particles

**DOI:** 10.3390/cells12020281

**Published:** 2023-01-11

**Authors:** Andi Alijagic, Nikolai Scherbak, Oleksandr Kotlyar, Patrik Karlsson, Xuying Wang, Inger Odnevall, Oldřich Benada, Ali Amiryousefi, Lena Andersson, Alexander Persson, Jenny Felth, Henrik Andersson, Maria Larsson, Alexander Hedbrant, Samira Salihovic, Tuulia Hyötyläinen, Dirk Repsilber, Eva Särndahl, Magnus Engwall

**Affiliations:** 1 Man-Technology-Environment Research Center (MTM), Örebro University, SE-701 82 Örebro, Sweden; 2 Inflammatory Response and Infection Susceptibility Centre (iRiSC), Faculty of Medicine and Health, Örebro University, SE-701 82 Örebro, Sweden; 3 Faculty of Medicine and Health, School of Medical Sciences, Örebro University, SE-701 82 Örebro, Sweden; 4 Centre for Applied Autonomous Sensor Systems (AASS), Mobile Robotics and Olfaction Lab (MRO), Örebro University, SE-701 82 Örebro, Sweden; 5 Department of Mechanical Engineering, Örebro University, SE-701 82 Örebro, Sweden; 6 KTH Royal Institute of Technology, Department of Chemistry, Division of Surface and Corrosion Science, SE-100 44 Stockholm, Sweden; 7 AIMES—Center for the Advancement of Integrated Medical and Engineering Sciences at Karolinska Institutet and KTH Royal Institute of Technology, SE-100 44 Stockholm, Sweden; 8 Department of Neuroscience, Karolinska Institutet, SE-171 77 Stockholm, Sweden; 9 Institute of Microbiology of the Czech Academy of Sciences, 140 00 Prague, Czech Republic; 10 Department of Occupational and Environmental Medicine, Örebro University Hospital, SE-701 85 Örebro, Sweden; 11 Uddeholms AB, SE-683 85 Hagfors, Sweden

**Keywords:** additive manufacturing, nanoparticle emissions, high-content screening (HCS), multivariate analysis, inflammation, targeted metabolomics, new approach methodologies (NAMs)

## Abstract

Additive manufacturing (AM) or industrial 3D printing uses cutting-edge technologies and materials to produce a variety of complex products. However, the effects of the unintentionally emitted AM (nano)particles (AMPs) on human cells following inhalation, require further investigations. The physicochemical characterization of the AMPs, extracted from the filter of a Laser Powder Bed Fusion (L-PBF) 3D printer of iron-based materials, disclosed their complexity, in terms of size, shape, and chemistry. Cell Painting, a high-content screening (HCS) assay, was used to detect the subtle morphological changes elicited by the AMPs at the single cell resolution. The profiling of the cell morphological phenotypes, disclosed prominent concentration-dependent effects on the cytoskeleton, mitochondria, and the membranous structures of the cell. Furthermore, lipidomics confirmed that the AMPs induced the extensive membrane remodeling in the lung epithelial and macrophage co-culture cell model. To further elucidate the biological mechanisms of action, the targeted metabolomics unveiled several inflammation-related metabolites regulating the cell response to the AMP exposure. Overall, the AMP exposure led to the internalization, oxidative stress, cytoskeleton disruption, mitochondrial activation, membrane remodeling, and metabolic reprogramming of the lung epithelial cells and macrophages. We propose the approach of integrating Cell Painting with metabolomics and lipidomics, as an advanced nanosafety methodology, increasing the ability to capture the cellular and molecular phenotypes and the relevant biological mechanisms to the (nano)particle exposure.

## 1. Introduction

Additive manufacturing (AM), also known as industrial three-dimensional (3D) printing, includes advanced manufacturing technologies that create solid objects of virtually any geometry. AM employs a laser or electron beam to fuse the polymeric, metallic, ceramic, or composite feedstock microparticle powders into desired products with a broad array of applications [1,2]. However, the advanced technologies and materials used in AM require effective approaches for proactive safety assessments, to limit the health risks associated with human exposure to unintentional (nano)particle emissions [3,4].

During open powder handling, printing, product post-processing, and machine cleaning, the feedstock and unintentionally formed micron- and nano-sized particles can, to a different extent, be emitted into the AM work environment. The existing air measurements reveal that different processes in AM entail the emission of large numbers of (nano)particles, reaching up to 500,000 particles/cm^3^ inside the printer hood. This means that AM workers risk to be daily and repeatedly exposed to (nano)particles with the unknown biological effects [5]. Hence, there is an increasing importance in investigating the ability of these (nano)particles, to induce the biological responses and to assess the possibilities for read-across with effects induced by other kinds of (nano)particles. In a recent study, we detected a limited toxicity in human lung and macrophage cell models exposed to condensate/spatter particles formed during the SLM printing of high Ni-containing alloys (Hastelloy X, Inconel IN939), and Ti6Al4V [6].

Since (nano)particles usually interact with multiple structures and biomolecules in the cells, and perturbate multiple biological pathways affecting the different cellular processes, it is usually difficult to deduce their complex mechanisms of action (MoAs) [7,8]. In order to scrutinize the (nano)particle toxicity in human cells and to obtain robust biomarkers of exposure, new approach methodologies (NAMs) show great promise [9]. Key categories within NAMs, include the in vitro high-throughput and high-content imaging assays along with in silico tools. Cell Painting is a high-throughput and high-content image-based assay that uses fluorescence cytochemistry and an automated image analysis to reveal the impact of the perturbation(s) on single-cell morphological phenotypes [10,11]. The morphology of cells closely mirrors its state, emerging from the dynamic functional requirements and thereby determines the future cell behavior [12,13]. The output results using Cell Painting have diverse applications, including the discovery of targets and the mapping of the compound bioactivity [14], the detailed study of MoAs, the compound clustering, based on MoAs [15], the discovery of unexpected MoAs [16], disease modelling [17], or the cell health phenotype prediction [18].

Cell Painting has so far mainly been employed to explore the effects of chemicals of both pharmaceutical and environmental relevance [15,19,20,21], leaving a large knowledge gap to be filled for its application in the (nano)particle safety assessment. In previous research, the integration of transcriptomics and Cell Painting data revealed that each data type provides the complimentary information for the induced MoAs [22]. In contrast to the gene expression, the metabolic profiles are not organism-specific and offer a great opportunity to profile the entire intracellular and extracellular metabolome [23]. Therefore, metabolomics can identify the early signs of altered cellular conditions that are crucial for elucidating the (nano)particle MoAs. Moreover, by providing mechanistic insights, the metabolomics data may also be used as biomarkers for biomonitoring purposes [24]. In addition, Cell Painting and omics data may support the identification of molecular initiating event(s) (MIE), as well as key event(s) (KE) that can be incorporated within the adverse outcome pathway (AOP) networks as next-generation standardized methodology approaches, to support the safety assessment of materials at the nanoscale [25].

The aim of this study was to assess the toxic effects of (nano)particles unintentionally emitted at metal AM occupational settings (henceforth “AMPs”) on human cells. Thus, we: (i) employed a range of physicochemical techniques to analyze the size, shape, bulk, and surface chemistry of AMPs collected in filters connected to a Laser Powder Bed Fusion (L-PBF) 3D printer, (ii) applied high-content screening (HCS) by using the Cell Painting assay to evaluate the subtle changes in the morphological profiles at the single-cell resolution, following the AMP exposure, supported by the analyses of the cell viability, generation of the reactive oxygen species (ROS), the AMP internalization, and the univariate and multivariate data analyses, and (iii) performed untargeted lipidomics and targeted metabolomics in a human lung epithelial and macrophage co-culture cell model, in order to scrutinize the effects of the AMPs at the level of metabolites, focusing on the inflammatory processes.

## 2. Results and Discussion

**Figure 1:**
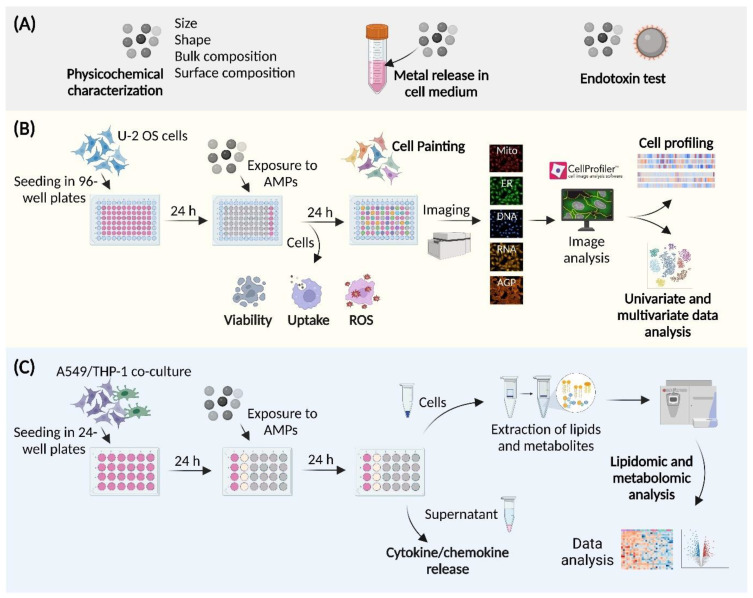
**Schematic overview of the experimental design.** To investigate the toxic effects of the (nano)particles unintentionally emitted at the metal AM occupational settings (AMPs), on human cells, the study was designed in three main phases. (**A**) Extensive AMPs’ physicochemical characterization, performed using a wide spectrum of analytical methods to support the data interpretation and comparability. In addition, the AMP dispersions were tested for the presence of the endotoxin. (**B**) Plate-based assays were employed to understand the AMP effects on the cell viability, the ROS production, and internalization. The high-content screening (HCS) by a Cell Painting assay, followed by the univariate, unsupervised multivariate, and supervised multivariate analyses, were performed to understand the impact of AMPs on the cells’ morphological profiles (U-2 OS cells). Mito—mitochondria; ER—endoplasmic reticulum; AGP—actin, Golgi, plasma membrane. (**C**) Lipidomic and metabolomic analyses were performed in a co-culture model (A549/THP-1 cells), mimicking the lung tissue as a potential key target organ for AMPs, and to close the knowledge gap on the AMP MoAs. Figure created with Biorender.com.

The experimental blueprint of this study was tailored, as summarized in Figure 1. In brief, the AMPs were: (A) characterized by a range of physicochemical methods, tested for the metal release in the cell medium, and for the endotoxin contamination, (B) analyzed by means of the Cell Painting assay, and (C) analyzed by the untargeted lipidomics and the targeted metabolomics, in order to understand their effects in the co-culture model of lung epithelial cells and macrophages.

### 2.1. Physicochemical Characterization, the Metal Release, and the Endotoxin Levels

The toxicological screening requires the collection of a large amount of airborne (nano)particles, usually up to milligram (mg) quantities [26]. This is challenging in an AM work setting, especially since the nano-sized particles have a negligible mass and the impactor samplers have technical limitations. An alternative approach involves the collection of particles from in-built filters within the AM printer. By using a straightforward and gentle mechanical scrapping of the AMPs adhering to the filter surface, we were able to collect sufficient amounts of AMPs, to execute the designated experiments. The filter had been in use for 2000 process-hours in a laser powder bed fusion (L-PBF) AM machine, using iron-based feedstock materials. The analysis of the physicochemical characteristics of the emitted (nano)particles, is of paramount importance, as the toxicity largely depends on factors, such as the particle size, chemical bulk and surface composition, shape, propensity to aggregate/agglomerate, and dissolution rate [27,28]. In addition, the physicochemical characteristics of the (nano)particles are key-governing factors when it comes to the interactions with biomolecules and to the processes taking place at the (nano)particle-cell interface [29,30].

**Figure 2:**
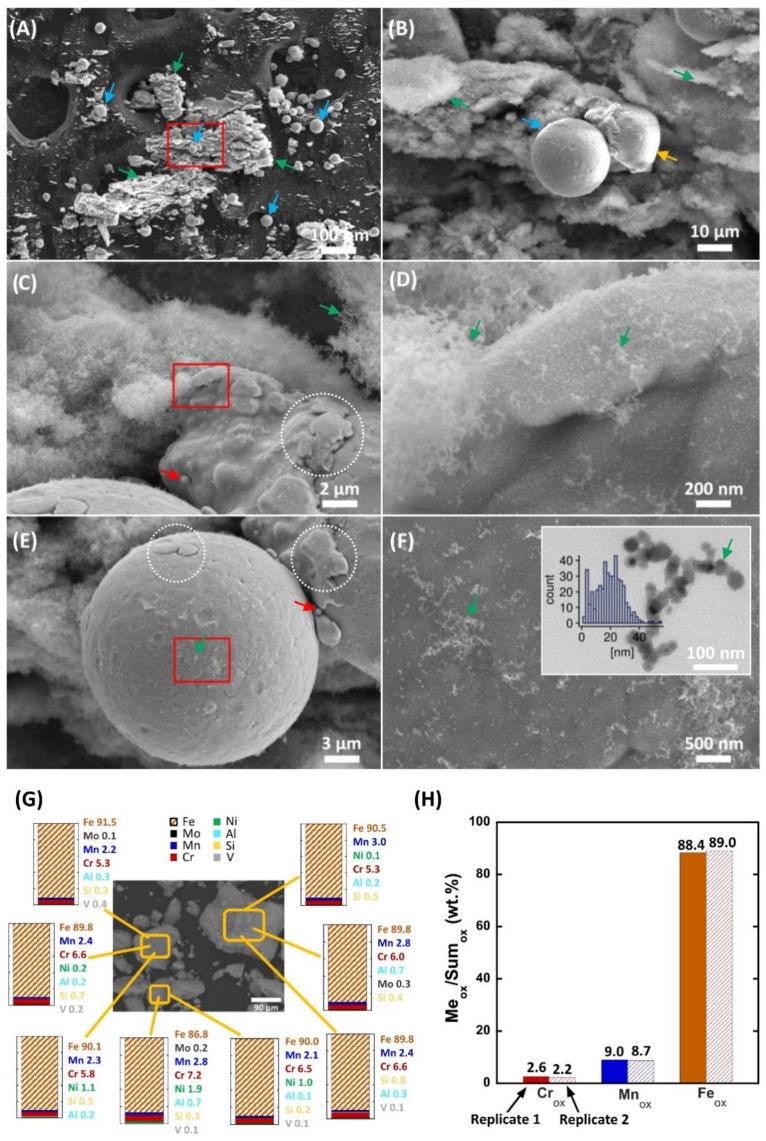
**Physicochemical characterization of the (nano)particles unintentionally emitted at the metal AM occupational settings (AMPs**). (**A**–**F**) Scanning electron microscopy (SEM) images demonstrating the size and shape characteristics of the collected AMPs. The blue arrows indicate the spherical micron-sized particles (possibly the feedstock material, based on size/shape/chemical composition), the yellow arrow highlights the micron-sized particles with an altered shape, and the green arrows indicate the large and irregularly shaped particle clusters. The red rectangular spaces in (**A**,**C**,**E**) are magnified in (**B**,**D**,**F**). The red arrows in (**C**,**E**) show the nanosatellites attached to the surface of the micron-sized particles. The white dashed areas in (**C**,**E**) demonstrate the alterations in the micron-sized particle surface topography. The green arrows in (**E**,**F**) show the tendency of the micron-sized particles to adhere to a large number of nanoparticle aggregates/agglomerates. The transmission electron microscopy (TEM) image and the graph in (**F**), upper right corner, report the nanoparticle size distribution and shape. (**G**) SEM combined with the energy dispersive spectroscopy (EDS) analysis of the bulk chemical composition (relative metal composition of Fe, Cr, Mn, Mo, Al, Si and V) of the AMPs (see also Figure S1). (**H**) X-ray photoelectron spectroscopy (XPS) analysis of the relative oxidized metal composition (Fe, Cr, Mn) of the outermost surface of the AMPs (both the micron-sized particles (surface) and the nanoparticles (surface/bulk)).

Electron microscopy images of the AMPs extracted from the filter cartridge are shown in Figure 2A–F. The results revealed that the AMPs were a complex mixture of spherical micron-sized particles (most probably feedstock material, based on size/shape/composition; Figure 2A,B, blue arrows), altered micron-sized particles (Figure 2B, yellow arrow), and large and irregularly shaped particle clusters (Figure 2A,B, green arrows). When observed under a higher magnification, it was evident that the clusters were composed of a large number of nanoparticles, most probably condensate/spatter particles generated during printing (Figure 2C,D, green arrows). These observations are in agreement with previous findings, in which the condensate particles have been described as the solidified particles, often in the nano-sized range, generated from the evaporation and condensation of metal alloy powders during the AM printing process [31,32,33,34]. The size of the spherical and altered micron-sized particles varied between 10 and 50 µm (Figure 2A,B), and revealed an altered surface topography (Figure 2C–E, white dashed areas), presence of nanosatellites (Figure 2C–E, red arrows), and the tendency to adsorb a large number of nanoparticle aggregates/agglomerates (Figure 2E,F, green arrows). Due to the resolution limitations of scanning electron microscopy (SEM), transmission electron microscopy (TEM) imaging was applied to further scrutinize the size and shape of the observed nanoparticles, after the sonication of the AMP dispersion, as this was performed prior to each cell exposure experiment. The TEM analysis disclosed that the size of the nanoparticles ranged from 2.41 to 55.1 nm with a mean size of 20.0 ± 9.13 nm (Figure 2F, size distribution graph), and that the nanoparticles mainly maintained a spherical shape and formed smaller aggregates/agglomerates, compared to the findings for the micron-sized particles (Figure 2F, upper right corner).

The chemical composition of the AMPs was assessed by means of SEM combined with energy dispersive spectroscopy (EDS). The results from examining eight random spots of different micron-sized particles, as the relative proportion of metals, excluding oxygen (O), are presented in Figure 2G. Iron (Fe) was the most common element in all particles; ranging between 86.8–91.5 wt.%. This is expected, since Fe-based steel feedstock powders were used in the AM printer of the study. The second most common metal was chromium (Cr); ranging between 5.3 and 7.2 wt.%, and manganese (Mn), as the third most common metal in an amount between 2.1 and 3.0 wt.%. The remaining metals were present, to a lesser extent, and included: molybdenum (Mo), aluminum (Al), nickel (Ni), silicon (Si), and vanadium (V), i.e., the typical alloy elements of tool steels. In summary, the alloying elements were present to a lower extent in the micron-sized particles, when compared with the nominal bulk content (see Table S1). The EDS mapping in Figure S1 outlined that the micron-sized particles also contained sulfur (S), while the nano-sized particles had a high oxygen content and contained Fe, Cr, Mn, Al, V, and Si (Figure S1).

X-ray photoelectron spectroscopy (XPS) was employed to examine the chemical composition of the outermost surface (5–10 nm) of the AMPs, i.e., reflecting the surface oxide of the micron-sized particles (Figure 2H). Due to the surface sensitivity of the technique, the signal originating from the nano-sized particles most probably reflected their bulk composition [35]. The results are presented as the mean values of both the micron- and nano-sized particles covering two separate 0.35 mm^2^ large surface areas. The main alloying elements of the printed alloys (Fe, Mn, Cr) were observed in the outermost surface and are presented in their oxidized states. Compared with the EDS relative compositional findings (Mn/Cr = 0.4 ± 0.1), the XPS results elucidated an almost 10 times higher fraction of oxidized Mn (mainly Mn (IV)), compared with oxidized Cr (as Cr(III)) (Mn/Cr = 3.5–4) in the Fe-rich surface oxide. None of the other tool steel alloy constituents (Mo, Al, Si, or V) was observed by means of XPS in the outermost surface.

The extent of the released metals from the AMPs upon incubation in the cell culture medium during the exposure to cells (24 h at 37 °C), was assessed and determined by means of atomic absorption spectroscopy (AAS). The results revealed minor amounts of Ni (<0.003% of the AMPs mass; <5 µg/L) and Mn (≈0.02%; 21 ± 4.5 µg/L) released into the cell medium after 24 h of exposure. These results are consistent with the XPS compositional analysis showing the oxidized Mn in the outermost (5–10 nm) surface oxide of the AMPs but no Ni (Figure 2H), i.e., localized at the interface towards the cells. The low level of the released Ni reflects the barrier properties of the surface oxides of the AMPs, similar to the previous findings, in the case of Ni-based condensate powders (Inconel IN939, Hastelloy-X, 18Ni300) and stainless steel (316 L) [6].

Finally, prior to the toxicological studies, the endotoxin content in the AMPs’ dispersions was analyzed. Endotoxin is a widespread contaminant originating from the Gram-negative bacterial cell wall and exhibit a great stability and broad distribution on all surfaces. Because the cells, especially the immune cells, are extremely sensitive to exceedingly low endotoxin concentrations (<20 pg/mL) [36], testing the AMPs’ dispersions for the presence of the endotoxin is essential to ensure that the possible effects on the cells are triggered by the (nano)particles themselves, and not by the undetected endotoxin contamination [37,38]. Using the chromogenic endotoxin quantification kit, the levels were quantified in the stock dispersion supernatant, as well as in the three highest AMP concentrations used in the toxicological assays. The investigations of the different (nano)particle concentrations are recommended, in order to evaluate and avoid interferences with the optical performance of the assay [39]. The level of the endotoxin in all tested samples was less than 0.01 EU/mL, considerably lower than reported values eliciting the unintentional immunological activation of the cells [36].

### 2.2. Cell Viability, Oxidative Stress, and the (Nano)Particle Internalization

**Figure 3:**
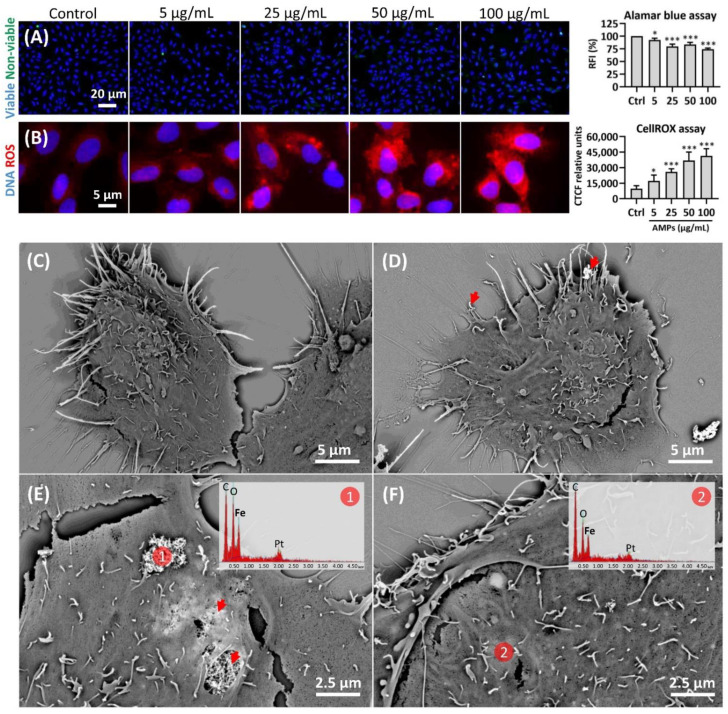
**Impact of the (nano)particles unintentionally emitted at the metal AM occupational settings (AMPs) on the cell viability/metabolic activity, ROS production, and internalization**. Plate-based assays with the U-2 OS cells exposed to a range of AMP concentrations (0–100 µg/mL) discloses that: (**A**) AMPs did not impair the cell viability (blue color—Hoechst 33342 labelling viable nuclei; green color—Image-iT DEAD Green viability stain labelling unviable nuclei), however, AMPs reduced the metabolic activity of cells, as shown in the Alamar blue assay (graph on the right). RFI—relative fluorescence units; (**B**) AMPs induced the ROS production and exerted oxidative stress in the exposed cells (blue color—Hoechst 33,342 nuclear labelling; red color—CellROX Deep Red ROS labelling). The graph on the right is a quantitative summary of the relative fluorescence obtained from the cells after the ROS labelling. The fluorescent signal was quantified using ImageJ software and the data is reported as the mean value of N = 150 cells, per condition. CTCF—corrected total cell fluorescence. * *p* < 0.05; *** *p* < 0.001. (**C**) Unexposed U-2 OS cells observed under the scanning electron microscope (SEM) at 10,000× magnification. (**D**–**F**) U-2 OS cells exposed to 50 µg/mL AMPs for 24 h and imaged under SEM. Red arrows indicate the AMP aggregates/agglomerates associated with the cell’s outer surface (**D**) or partially covered by the cell membrane (**E**). Energy dispersive spectroscopy (EDS) spectra in the upper right corners (**E**,**F**) indicate the composition of the electron-dense AMP areas. Pt—platinum.

Prior to the Cell Painting, we examined whether the AMPs would: (i) impact the U-2 OS cell viability, metabolic activity, and cell number, (ii) induce oxidative stress, and (iii) become internalized by the cells. The initial analysis of the cell viability is vital because the highly cytotoxic (nano)particle concentrations should be avoided, as the apoptotic/necrotic cells provide only limited information on the (nano)particle MoAs in the cells [8], which are the aspects in focus for the Cell Painting and omics studies. Using a HCS LIVE/DEAD assay, no evident effect of the AMPs on the cell viability was detected within the studied concentrations (5–100 µg/mL) and in the frame of the selected exposure time (Figure 3A). However, the Alamar blue assay disclosed that the metabolic activity of the cells was reduced by up to 25% by the highest AMP concentration (Figure 3A, right panel). Th reduced metabolic activity may be attributed to the reduced cell proliferation rate, due to the exposure, and not to the cytotoxicity per se, as discussed by Longhin et al. [40]. This was further corroborated by the CellProfiler nuclei counts revealing the decreased cell numbers of up to 24% for the highest AMP concentration (data not shown).

The CellROX assay disclosed the induction of the reactive oxygen species (ROS), following the 24 h exposure to a range of AMP concentrations (Figure 3B). Even the lowest tested AMP concentration (5 µg/mL) triggered a significantly increased ROS production, when compared to the control. Moreover, at 50 µg/mL, the cell responses reached a plateau, maintaining a similar ROS level also for the AMPs at a (nano)particle concentration of 100 µg/mL (Figure 3C). Alternatively, at the highest concentration (100 µg/mL), the cells could be saturated with AMPs and therefore would not take up additional (nano)particles. This is in agreement with previous studies, showing the ROS production as one of the main mechanisms for the (nano)particle-induced cell injury, also in the case of, e.g., Fe-, Ni-, and Co-based nanoparticles [41,42,43]. The ROS can, for instance, be generated directly from the free radicals on the (nano)particle surface or via the transition metal (nano)particles, such as iron (in particular hydroxyl radicals), by acting as catalysts in the Fenton-type reactions [44]. The release of Mn from the AMPs during incubation with the cells, in this study, may be one of the underlying mechanisms that induces the ROS formation and oxidative stress. This is in line with the reported findings using the ToxTracker assay [45,46], for which the Mn and Mn_3_O_4_ nanoparticles induced the oxidative stress marker Srxn1 [47]. Recent findings also show that Mn stimulates the mitochondrial H_2_O_2_ production over the entire range of concentrations, from those required for the normal physiology to the concentrations causing cell death [48,49]. Even though Mn has been found to induce adverse effects, it is an essential metal for human health and specific concentrations are required to maintain different cellular processes regulating, for example, oxidative stress [50]. In addition, the ROS formation is a very common MIE, in the context of AOP networks, since the cells try to neutralize the foreign (nano)particles and, as a result, generate high amounts of ROS [51]. Consequently, the ROS induces very common KEs—the oxidative stress and activation of the oxidative stress pathways. Finally, it is important to emphasize that the (nano)particles may interfere in a number of ways with the ROS detection assays [52,53]. Therefore, it is crucial to design well-defined methods that will affirm the ROS production, following the exposure to (nano)particles [54].

In order to further analyze the (nano)particle-cellular interactions, including the potential internalization, the U-2 OS cells exposed to AMPs (50 µg/mL) for 24 h were analyzed by SEM, coupled to EDS (Figure 3C–F). The results revealed that the AMP aggregates/agglomerates that were associated with the cells (see Figure 3D,E, red arrows), ranged in size from <1 to about 10 μm in diameter. Notably, the back-scattered electron SEM permitted imaging that could discriminate both the organic (dull shine) and inorganic (intense brightness) materials inside of the cells. The area labelled with number one (Figure 3E) indicates the AMP aggregate/agglomerate associated with the outer cell membrane, while the arrows indicate the aggregates/agglomerates partially covered by the cell membrane. The area labelled with number two (Figure 3F) displays the AMP aggregate/agglomerate fully internalized by the cell. The composition of the electron-dense AMP areas, located both outside and inside the cells, was confirmed by the means of EDS (Figure 3E,F, see EDS spectra). The evaluation of the (nano)particle internalization is a highly important step prior to the HCS, as it gives the crucial information on whether the observed effects ultimately arise from the external interactions at the (nano)particle-membrane interface, from the intracellular interactions between the (nano)particles and the specific cell compartments, or from a combination of the external and intracellular events. Importantly, the size distribution of the AMPs was very broad, and further experiments beyond the scope of this study would be needed to outline the actual mechanisms of endocytosis, as well as to investigate the impact of the AMP size on the whole process in a time-resolved manner. Thus, we envision future studies focusing solely on the internalization mechanisms for AMPs by using living cells and various types of microscopy techniques with a high spatial and temporal resolution [55].

### 2.3. Profiling of the Cell’s Morphological Phenotypes by the Cell Painting Assay

In order to investigate the effect of the AMPs on the single-cell morphological phenotypes, the HCS by the Cell Painting assay was employed [11]. To the best of our knowledge, this is the first attempt to use this non-target high-throughput image-based assay, to reveal the effects of the (nano)particle exposure on the different cell compartments/organelles. For that scope, we used the U-2 OS cell line, that is widely used in the HCS, because the individual cells grow in a manner conducive to the fluorescent imaging and analysis [11]. Since the Cell Painting assay is intended to be unbiased and not targeted to a particular biological area or effect of interest, the use of a well-established and optimized cell model, such as the U-2 OS cells, is sensible. This cell line has also proven to be very useful in other studies of morphological profiling, mainly for pharmaceuticals and environmental chemicals [15,19,21]. In the morphological profiling, it is also anticipated that several very specific biological effects and pathways can be interrogated, even in a relatively “generic” cell type [56,57].

In short, the cells were exposed for 24 h to nine different AMP concentrations (0.156–100 µg/mL). In addition to the AMP concentrations investigated for the cell viability and ROS assay, additional lower concentrations (0.156–2.5 µg/mL) were tested to evaluate whether the Cell Painting can detect the subtle changes in the cell morphological phenotypes, even at very low and occupationally relevant AMP concentrations. Even though the AMPs may not be cytotoxic at these concentrations, the information on the altered morphological phenotypes could be useful as early non-target biomarkers when examining the effects of (nano)particles on multiple human-derived cell types, to predict the functional impact, particularly at the single-cell level [58]. Since we were mostly interested in the primary effects of AMPs, the exposure time was chosen, based on the findings that the prolonged exposure (longer than 24 h) may lead to secondary effects (e.g., changes in the DNA morphology) [15]. Cell Painting was performed using a set of fluorescent probes and fluorophore-conjugated small molecules, followed by high-content imaging, image analysis using CellProfiler version 4.2.1, and the data analysis of the morphological features (see workflow in Figure 1B). To check whether AMPs interacted with the Cell Painting fluorescent probes and/or display autofluorescence, the unstained AMPs and AMPs after staining with the Cell Painting stain cocktail, were imaged. In both cases, no detectable autofluorescence was observed (data not shown).

**Figure 4:**
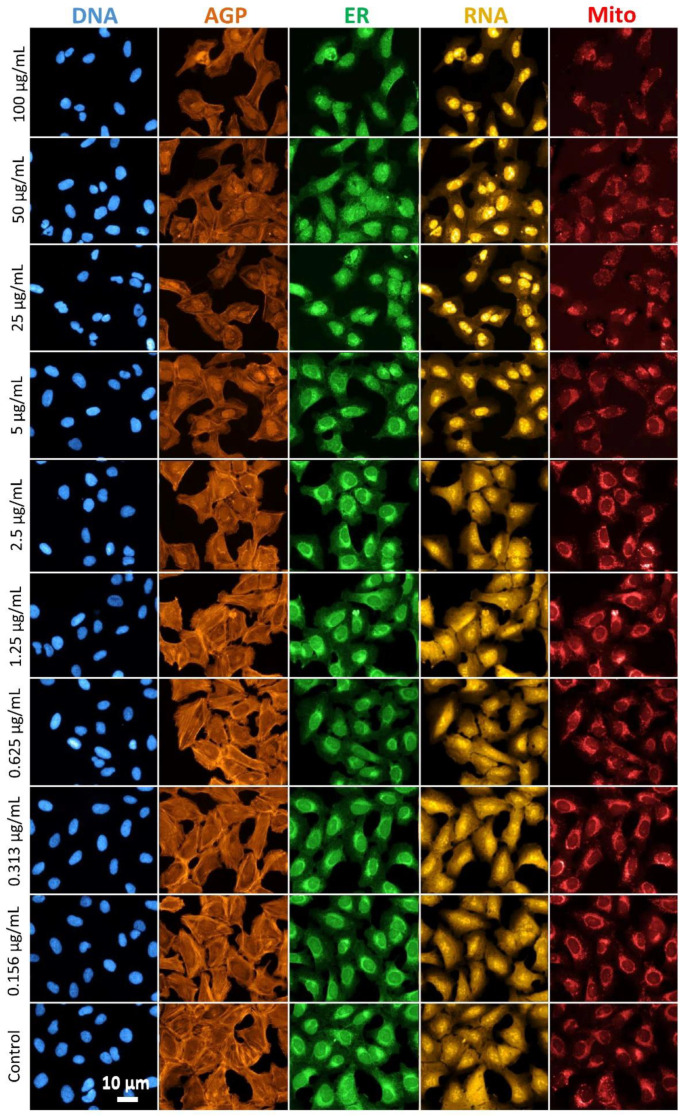
**Cell Painting labelling patterns in the U-2 OS cells.** Representative images of the control and cells exposed to 0.156, 0.313, 0.625, 1.25, 2.5, 5, 25, 50, and 100 µg/mL of the (nano)particles unintentionally emitted at the metal AM occupational settings (AMPs), live-labeled for the mitochondria (Mito), fixed, permeabilized, and labeled with the remaining fluorescent probes for the nuclei (DNA), actin/Golgi/plasma membrane (AGP), endoplasmic reticulum (ER), and RNA/nucleoli (RNA). Distinct morphological effects of the AMPs observed qualitatively, are evident in each channel, with the exception of the DNA-related features, where the morphological changes can be observed only to a lower extent. All images were acquired at a 20× magnification.

For all of the tested AMP concentrations, the visual changes in the cell morphological phenotypes were obvious, and mainly consistent with changes in the (nano)particle concentration. The effects of exposure to the morphological profiles of the U-2 OS cells are presented in Figure 4 and show the differential effects of AMPs on the eight cell compartments investigated, including nuclei (DNA), actin/Golgi/plasma membrane (AGP), endoplasmic reticulum (ER), RNA/nucleoli (RNA), and mitochondria (Mito).

The morphological patterns in the AGP channel showed the highest degree of qualitative dissimilarity among the different AMP concentrations investigated, with a clear concentration-dependent response (Figure 4, AGP). The prominent fluorescent labeling of the cytoskeleton (visualized as striations) was observed in the control cells. However, with an increased AMP concentration, the labeling in the cytoplasm appeared less intense, especially for the four highest concentrations. The prominence of the plasma membrane labeling was also reduced, as the AMP concentration increased. We hypothesize that the main mechanisms behind the observed cytoskeleton alterations were governed through the interconnection of the AMPs internalization and the ROS formation, as demonstrated in Figure 3. The actin-dependent endocytosis has been previously proposed as a major internalization mechanism for the numerous (nano)particles in the different cell types [59]. Concurrently, the (nano)particles act through the ROS formation to disrupt the actin dynamics, which in turn alters the cytoskeleton and impacts the cell morphology [60,61]. The common mechanism that the nanoparticles use to disrupt the cytoskeleton is the depolymerization of F-actin [62], which in turn may lead to the reduced staining efficiency of phallodidin that binds specifically at the interface between the F-actin subunits [63].

Based on the microscopic examination, the fluorescence signal of the ER decreased, especially for the three highest particle concentrations (100, 50, and 25 µg/mL), while the lower concentrations (<5 µg/mL) even tended to increase the fluorescence signal of the ER, located mainly in the perinuclear space (Figure 4). The ER structure was labelled by concanavalin A, which selectively binds to the alpha-mannopyranosyl and alpha-glucopyranosyl residues in glycoproteins found in the ER membranes. Thus, decreased staining efficiency of concanavalin A may indicate the AMP impact on the integrity of the ER membranes. Zhang et al. [64] showed that exposure to Fe_3_O_4_ led to the disrupted ER vesicles in the MCF-7 cells. However, the AMP effects on the ER require further inquiry and it is worth considering by replacing one or more of the original stains for a more targeted one, in order to investigate the mechanisms behind it. The exposure to the AMPs altered the distribution of the cytoplasmic RNA with a shift from the cytoplasm to the nucleus. The nucleoli became highly defined and more intensely stained with the increased concentration of AMPs (Figure 4).

The mitochondria (Mito) likewise had an asymmetric perinuclear localization, with two distinct trends of morphological changes, as the result of the AMP exposure. While the highest concentration (100 µg/mL) reduced the MitoTracker staining efficiency, all of the lower AMP concentrations displayed increased mitochondrial morphological profiles, as compared to the unexposed control cells. We hypothesize that the observed decrease of the fluorescence in this channel at the highest tested AMP concentration was due to the elevated ROS level causing the disruption of the mitochondrial inner-membrane permeability and the collapse of the membrane potential [65,66,67]. The described events will lead to the subsequent water accumulation, thus causing the mitochondrial matrix to swell. Under these circumstances, the mitochondria are more transparent, and hence the signals collected by the instrument decrease [67]. These findings also have an importance when it comes to the organelle KEs, since the impairment of the mitochondrial membrane potential can lead to the mitochondrial dysfunction as a critical KE, ultimately causing acute toxicity.

Qualitatively, the DNA compartment was the least affected, as compared to other compartments. This is in agreement with the literature findings, where it is generally assumed that the (nano)particles, as physical objects and especially when aggregated/agglomerated, cannot easily penetrate the nucleus and cause structural damage [68]. Moreover, a study by Ahlinder et al. [69] reports the presence of the Fe nanoparticles in the nucleus, although without shedding light on the nuclear internalization mechanisms.

**Figure 5:**
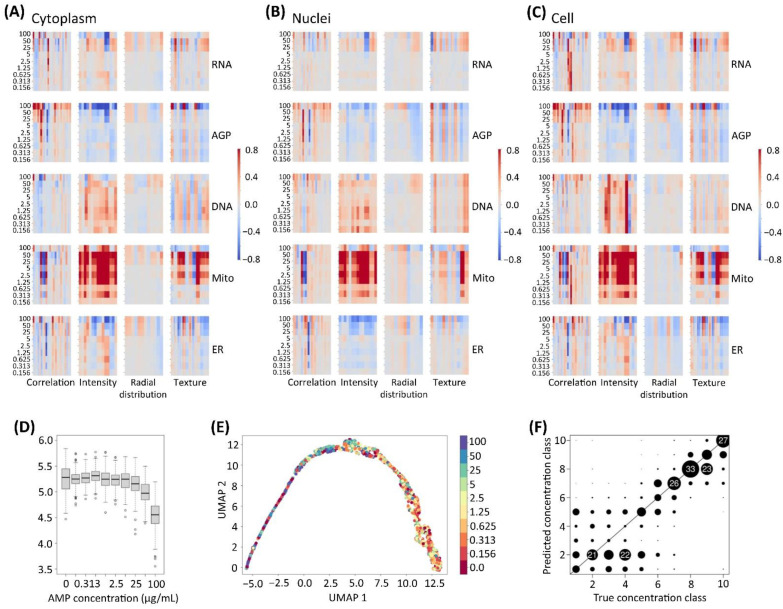
**Quantitative summary of the morphological effects for the (nano)particles unintentionally emitted at the metal AM occupational settings (AMPs).** Treatment-level feature data were normalized and scaled per plate (N = 3), the batch effect was corrected, and then averaged. Three heatmaps were established, corresponding to the morphological features organized by compartment: (**A**) cytoplasm (962 features), (**B**) nuclei (976 features), and (**C**) cell (998 features); by feature group: correlation, intensity, radial distribution, and texture; and by fluorescent channel: nuclei (DNA), actin cytoskeleton/Golgi/plasma membrane (AGP), endoplasmic reticulum (ER), RNA/nucleoli (RNA), and mitochondria (Mito). The colors represent the fold change in each measured feature, with respect to the unexposed control cells. The rows correspond to the individual concentrations of the AMPs (0.156–100 µg/mL). Exposure concentrations are in descending order from top to bottom. Columns represent the individual morphological features. Data were derived from 435,678 single cell profiles distributed across six technical replicate wells of three microplates/biological replicates (N = 18 wells in total). (**D**) Boxplots for the feature *Texture_Entropy_AGP_5_02_256* are shown as an example of a feature that is clearly dependent on the AMP concentration (*x*-axis). (**E**) Uniform manifold approximation and projection (UMAP) was employed at the image level on the median of the morphological profiles of the U-2 OS cells exposed to the AMPs. Color legend indicates the AMP concentrations (µg/mL). (**F**) Results for the sparse partial least squares discriminant analysis (sPLS-DA) predictions averaged over three cross-validation runs, as a scatterplot. Predicted concentration class (*y*-axis) is shown as dependent on the true concentration class (*x*-axis). Circle radius visualizes the frequencies for the respective result, with numbers as text for values larger than 20. Sensitivity for the predictions of large AMP concentrations was 82%, the specificity was 94%. Concentrations of AMPs were considered “small” if less than, or equal to 1.25 µg/mL. Clear concentration dependency, with the predictive potential for the new samples, could be found for the AMP concentrations larger than 2.5 µg/mL.

To more easily identify the patterns of the morphological effects in the Cell Painting data across the nine AMP concentrations tested, a heatmap was constructed that illustrated the normalized effect magnitudes of the AMP exposure, per compartment, including the cell, cytoplasm, and nuclei. The heatmaps were constructed for every channel, including DNA, AGP, ER, RNA, and Mito. The features were grouped into several categories, including the correlation, intensity, radial distribution, and texture (Figure 5A–C).

Overall, the heatmaps for the AMP exposure indicated that a large number of features followed a concentration-dependent response pattern, especially in the case of the AGP, ER, and Mito profiles. Based on the data obtained using the CellProfiler, a variety of features related to the intensity and texture of the AGP and ER was sharply reduced, as also revealed in the high-content images (Figure 4). Interestingly, the AMP exposure led to the reduction in a set of AGP features within the radial distribution group (see Figure 5B), possibly indicating that the AMPs induced the shrinkage of the nucleoskeleton, due to its intracellular accumulation. The exposure to the AMP concentrations of less than, or equal to, 5 µg/mL, induced an increase in a smaller set of features related to the intensity of the ER, following the trend of the increased fluorescence observed in the high-content images (Figure 4, ER channel). Moreover, the AMP exposure resulted in a strong increase of the intensity and texture features of the mitochondria. The pronounced changes in the mitochondria are probably a consequence of oxidative stress induced by the exposure (as shown in Figure 3B). Even trace amounts of the ROS are capable to strongly activate the mitochondria [70]. Collectively, the impact of the AMPs on the AGP, ER, and Mito features agrees with a previous report stating that the iron-oxide nanoparticles induce the autophagosome accumulation via the mitochondrial damage and by the ER and Golgi apparatus stress [64]. In addition, the subsets of the features related to the staining intensity and the texture of DNA, were also affected, even at very low concentrations. This observation demonstrates the high sensitivity of the Cell Painting assay to detect very subtle changes that were undetectable in the fluorescent images, per se. The RNA features related to the intensity, texture, and radial distribution, were affected in the (nano)particle concentrations exceeding 5 µg/mL. In addition, the changes in the correlation group, which measures the correlation between the intensities in the different images (e.g., different channels), were observed for a number of morphological features in each cell compartment.

In order to check whether the cell morphological phenotypes depend on the physicochemical characteristics of the (nano)particles, the cells exposed for 24 h to SiO_2_ particles, a known inflammation inducer, were profiled and compared with the results of the AMP-exposed cells. The heatmaps per cell, cytoplasm, and nuclei compartment are visualized in Figure S2. The SiO_2_ particles induced the reduction in the cell viability (data not shown) and the concentration-dependent morphological changes at a higher fold change; the results substantially differed, when compared to the cells exposed to AMPs. The SiO_2_ particles strongly reduced the staining intensity of RNA, reduced the texture, and increased the staining intensity for several ER features in the cytoplasm and cell compartments. In addition, the SiO_2_ exposure caused a prominent increase in a subset of DNA intensity features, as well as induced a large impact on the nucleoskeleton (see Figure S2, nuclei, DNA, and AGP channels). When compared to the AMPs, the SiO_2_ particles also caused more potent effects on the radial distribution of the RNA and AGP features in the cytoplasm and cell. Taken together, these data suggest that changes in the cell morphological phenotypes detected by the Cell Painting assay depend on the physicochemical characteristics of the (nano)particles.

### 2.4. Biological Implications of the Cell Painting Features

Apart from the general trends, the changes in a number of individual features were identified, and their biological implications were further scrutinized. The increased activity between the ER and Golgi apparatus, as observed in the feature *Cells Correlation_Overlap_AGP_ER*, indicates a possible increase in the lysosomal activity, as recently hypothesized by Seal et al. [71]. The nanoparticles typically accumulate in lysosomes, and the impairment of the lysosome homeostasis has been proposed as an emerging mechanism of nanotoxicity [72]. Another feature that was found altered is *Cells Intensity_MaxIntensityEdge_AGP*. The edge of the segmented object potentially indicates the loss of the membrane integrity [71]. The nanoparticles are well known to interact strongly with the cell membrane, via electrostatic and non-electrostatic interactions, leading to changes in the membrane integrity and fluidity [73,74]. Moreover, the decrease of the feature *Cells Texture_Entropy_AGP_3_01_256* relates to the texture of the stain and might indicate the disruption of the actin network. Interestingly, the AMP exposure reduced the feature *Cells Neighbors_PercentTouching_5*, indicating the possible formation of intercellular gaps. This agrees with a previous finding suggesting that the nanoparticle exposure perturbs the actin networks and reduces the area of the cell-cell junctions [75].

In addition, the features related to location and granularity were analyzed. Due to the low variations in these feature groups upon the AMP exposure, they were excluded from the final heatmaps. However, it is interesting to mention the increase in the features *Cytoplasm Granularity_13_Mito* and *Cytoplasm Granularity_14_ER* in the AMP-exposed cells; the features that are probably indicating an ongoing ER stress and the possible fragmentation of the ER and the mitochondrial membrane [71]. This hypothesis is further corroborated by the change in the feature *Cytoplasm Texture_AngularSecondMoment_ER_10_00_256,* confirming that the AMPs affected the ER evenly. The texture is related to the topography of the cells and fewer structures are present, the topographical landscape of the cells is more even. The alteration of the feature *Cytoplasm Intensity_MaxIntensityEdge_Mito* most probably unveils the loss of the integrity of the mitochondrial membrane. Moreover, a change in the feature *Cytoplasm RadialDistribution_MeanFrac_mito_tubeness_3of16* discloses the effect of the AMPs on the mitochondrial shape, and it is well-known that the depolarized mitochondria undergo inner membrane rounding, resulting in the shape deformation [76,77].

### 2.5. Curation Strategies for the Cell Painting Datasets

In order to determine whether the AMPs affected the cell morphology in a concentration-dependent manner, the Cell Painting features were analyzed using univariate statistics. The ANCOVA models were run for each of the 977 image-based features, to establish whether a feature would exhibit a significant correlation with the concentrations of the AMPs. The plate batch effects were accounted for in these models, and 783 (80% of all analyzed features) were found to have a significant correlation with the AMP concentration (47% positive and 53% negative correlations) on a significance level of 5%, when using the Bonferroni correction for multiple testing. These results convey that the AMPs had a major effect on the cell morphology, and in a concentration-dependent manner, the AMPs impacted on the majority of the Cell Painting features on the image-level, as the averaged values of the different features for all cells within one image. As an example of the AMP concentration dependency, one of the strongly significant Cell Painting features, *Texture_Entropy_AGP_5_02_256* is presented in Figure 5D, showing a clear response for the higher (nano)particle concentrations.

To interpret the full extent of the high-content imaging data generated in the Cell Painting and to further examine the correlation between the changes of the morphological features and the AMP concentration, the dimensionality of the Cell Painting datasets was reduced using the uniform manifold approximation and projection (UMAP) [78]. The UMAP embedding results were analyzed for the batch effect corrected data. The scatterplot for the first two components with obvious tendencies to mirror the AMP concentration-related gradient is presented in Figure 5E. The lower concentrations (darker colors) were predominant for the left part of the UMAP profile, whereas the higher concentrations (lighter colors) were more frequently observed for the samples displayed in the right part. Even though many samples with deviating concentrations were detected, the general tendency can still be observed. This means, that even for an unsupervised multivariate method, the AMP concentration is a dominant driver of the overall morphological effects observed in the cells. We envision that the dimensionality reduction of the large Cell Painting datasets will be helpful in predicting similar MoAs in future investigations, and that such main components of the overall Cell Painting features could help in the classification of the (nano)particles, based on their hazardous potential. A similar approach was recently applied by Rietdijk et al. [21] to examine which constituent of the chemical mixture predominately elicits the effects in different human cell models.

To explore whether the AMP concentrations could be predicted by the Cell Painting features, a supervised analysis was employed by means of a sparse partial least squares discriminant analysis (sPLS-DA) approach. The Cell Painting results from two of the three microplate results served as the training data, whereas the remaining microplate was left out. The average results for the prediction of the concentration of the left-out test data (the microplate was not used for the training) are visualized in Figure 5F. The predicted higher concentrations of AMPs corresponded to the truly high concentrations with a high sensitivity (86% ± 7), specificity (93% ± 3), and balanced accuracy (90% ± 4) for the test data. The imported variables chosen by the L1-penalisation approach in this specific sPLS-DA method, for the three cross-validation runs, resulted in an average of 426 selected features for each run and an overlap of 251 selected features. Hence, the Cell Painting features could be used to predict the concentrations of AMPs with an expected high accuracy, especially for even the detailed differences in the concentration, for the larger concentrations of AMPs.

In summary, using the Cell Painting assay and measuring the single-cell morphologies, the distinct (nano)particle-specific morphological changes and the early non-target phenotypic markers were identified upon exposure to a wide range of AMP concentrations, including environmentally-relevant concentrations (e.g., 0.156 µg/mL). The identification of the distinct cell morphological phenotypes following the exposure, demonstrated that the Cell Painting holds an excellent potential as a NAM for the quantitative analysis of complex events and for the visualization of the relevant morphological phenotypes, with a single-cell resolution. This facilitates the identification of the potential risks and enhances the traceability of the threats that unintentionally released (nano)particles may pose, from an occupational safety perspective. Moreover, while classical in vitro assays (e.g., viability, ROS, and genotoxicity assays, etc.) focus on the single endpoints and unveil the (nano)particle effects, mainly at toxic levels, the Cell Painting assay discloses subtle morphological alterations before the exposure reaches toxic levels. Moreover, as an untargeted assay, the Cell Painting provides tools to study multiple cell responses, simultaneously [21]. In line, analyses of the Cell Painting profiles have earlier been found to be predictive for various cell health outcomes, such as proliferation, cell death, oxidative stress, genotoxicity, cytotoxicity, and cell cycle phase [79,80]. Even if the Cell Painting, at its best, can predict the MoAs used by the (nano)particles to exert the adverse effects, the omics technologies can further scrutinize the molecular targets within the MoA and enhance the output by the detailed mapping of the cellular and molecular phenotypes induced upon the exposure to the (nano)particles.

### 2.6. Lipidomics

**Figure 6:**
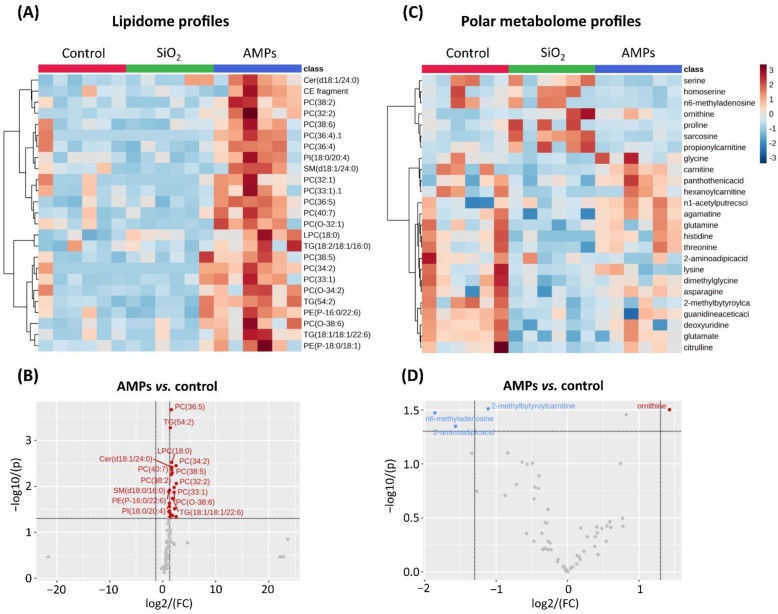
**Effects of the (nano)particles unintentionally emitted at the metal AM occupational settings (AMPs) on the lipidome and polar metabolome**. The fold changes (blue and red) of the top 25 out of 73 identified lipids, via the untargeted lipidomics (**A**) and the top 25 out of 63 identified polar metabolites, via the targeted metabolomics (**C**) are represented as feature-clustered heatmaps. Each column within the heatmaps represents one of three biological replicates with two technical repetitions. SiO_2_ was used as a positive control. Volcano plots show the up-regulation (red) or down-regulation (blue) of the lipids (**B**) and polar metabolites (**D**) after 24 h of exposure to 25 μg/mL AMPs. The log2 (FC—fold change) of the relative abundance of the lipids/polar metabolites in the AMP-exposed cells and in the control cells. The *y*-axis represents the −log10 (*p*-value) between the exposed and control samples. Results reported in the volcano plots (**B**,**D**) are a summary of three biological replicates with two technical repetitions.

The large surface area of the (nano)particles increases the possibility for interactions between the particles and cellular membranes. In accordance, the Cell Painting assay revealed that the AMP exposure significantly impacted the Golgi/plasma membrane and the ER compartments, both membranous and lipid-rich cell structures. Lipids are a multifunctional class of metabolites with numerous structural and signaling functions, including the actin assembly and the recruitment of molecular motors to the sites of ingestion [81]. Previous studies identified several lipids as markers of different cellular stress conditions, and further provided key information on the biochemical pathways involved in the cellular processes [82,83]. The lipid remodeling and changes in the lipid metabolism are essential for mounting an effective inflammatory response, they are involved in the regulation and fine-tuning of its course and cessation [84,85]. Therefore, we envisioned that the variations in the lipid composition could unveil the MoA of the AMPs and provide the metabolite markers for the AMP-induced adversity. Hence, to further dissect whether the AMPs would affect the cellular lipidomic profiles, an A549/THP-1 co-culture model was constructed, in which the cells were exposed for 24 h to 25 µg/mL of AMPs (non-toxic concentration, based on the Alamar blue assay; Figure S3) or 70 µg/mL of the SiO_2_ particles, the latter as a potent inducer of inflammation (positive control). The cellular model was used as a proxy to the human respiratory tissue, which is the main target of the (nano)particles, following inhalation [86]. The collected cell pellets were analyzed by means of a lipidomic analysis. An overview of the lipid changes across the AMPs and the SiO_2_-exposed cells is given in Figure 6A.

The final lipidomic datasets used for the analysis included a total of 73 identified lipids, and the data are reported in a feature-clustered heatmap showing the changes in the lipid profiles dependent on the AMP exposure. The dataset was further analyzed using ANOVA to determine the lipids that showed significant changes at the 95% confidence level with a post hoc Tukey honest significant difference (HSD) test. Observing the cumulative number of changing lipids across all samples, using the control as a reference point, a total of 13 lipids changed (*p* < 0.05), following the exposure to the AMPs and SiO_2_, respectively. The individual lipids with significant changes were clustered into various lipid classes, including (lyso)phosphatidylcholines (LPC): LPC(18:0); phosphatidylcholines (PC): PC(32:1), PC(33:1), PC(34:2), PC(36:4), PC(36:5), PC(40:7), PC(O-32:1), and PC(O-38:6); phosphatidylethanolamine (PE): PE(P-18:O/18:1); phosphatidylinositol (PI): PI(18:0/20:4); sphingomyelins (SM): SM(d18:1/24:0), and SM(d36:1). Overall, 24 lipids (Figure 6B, volcano plot) displayed the significant up-regulation (*p* < 0.05 and fold change of >2) after exposure to 25 μg/mL AMPs. In addition, the SiO_2_ particles mediated a quite opposite effect on the lipidomic profile, down-regulating the levels of PC(O-32:1) and SM(d18:1/24:0) (Figure S4).

The accumulation of triacyclglycerols (TGs), TG(54:2) and TG(18:1/18:1;22:6) was observed, following the exposure to 25 µg/mL AMPs (Figure 6B). This is consistent with the increased ROS production (observed in Figure 3B), and may be explained by the TGs being mostly non-membrane associated, and have been found increased, due to the oxidative stress induced by the exposure to metal oxide nanoparticles, such as aluminum oxide and titanium dioxide [87,88]. In parallel, the analysis revealed a large increase in the membrane lipids, including the predominantly PCs, ceramides (Cer), LPC, PI, and SM. Of note, a large part of these lipids were polyunsaturated fatty acyls (PUFA) containing lipids that have been found to increase the membrane fluidity [89]. The observed changes in the composition of the membrane lipids could therefore suggest the membrane remodeling, which could be further linked to the mechanism of the AMP internalization. Moreover, the PIs are central to the cytoskeletal rearrangements that underlie the engulfment of the particulate matter [81]. The potential membrane remodeling seems to be (nano)particle-specific because, in contrast to the observations made for the AMPs of this study, the copper oxide nanoparticles tend to increase the accumulation of ceramides and to reduce the cell viability [83]. The combination of the Cell Painting and lipidomics showed that the lipidomic changes and the morphological effects co-occur and appear to be driven by the exposure to AMPs. However, further integration of the Cell Painting and omics data will be the topic in future studies.

### 2.7. Targeted Metabolomics

Following the observation with the changes in the lipids induced by the exposure to the AMPs, our untargeted lipidomics approach was complemented by the targeted metabolomic analysis of the selected polar metabolites closely linked with inflammation. By this, we aimed to discern whether the AMP-induced oxidative stress and membrane remodeling can promote inflammation and reduce the knowledge gap on how the AMPs interact with cells. The interactions between the (nano)particles and the biological entities (e.g., cell receptors, membranes) usually lead to the innate immune reactions, involving the metabolic reprogramming. The immune reactivity results either in the immune tolerance, an active response that becomes part of the immune memory, or in the uncalibrated and long-lasting inflammation leading to the pathological consequences [90,91,92]. Thus, under the same exposure conditions described in the previous section, the A549/THP-1 cells were exposed to AMPs and analyzed for the polar metabolite profiles.

A total of 63 polar metabolites, closely related to the inflammatory responses in cells, were determined and their profiles are summarized in the feature-clustered heatmap (Figure 6C). In contrast to lipidomic profiles, the cumulative analysis did not reveal any significant variations in the levels of the metabolites among the various sample groups. However, several metabolites showed a trend of reduced concentrations in the AMP-exposed cells, whereas the SiO_2_ particles induced the very distinct metabolite profile, when compared to the AMPs or controls. To further scrutinize the effects of the AMPs, a volcano plot was constructed to compare how many metabolites displayed a significant change (*p* < 0.05 and fold change of >2) versus the control cells (Figure 6D, volcano plot). The results revealed that three metabolites were down-regulated (n6-methyladenosine, 2-aminoadipicacid, and 2-methylbutyroylcarnitine), and one was up-regulated (ornithine).

N6-methyladenosine is the most prevalent internal mRNA modificator [93]. Recent studies have shown that the n6-methyladenosine RNA epigenetic modification plays important roles in regulating the inflammatory reactions [94]. A study by Gu et al. [95], demonstrated that the inhibition of the n6-methyladenosine mRNA modification constrain the M1 and M2 macrophage polarization, simultaneously. Ornithine, moreover, is involved in the regulation of the macrophage functions, and is transformed by ornithine decarboxylase (ODC), which limits the M1 macrophage activation [96]. Moreover, the cross-analysis of these metabolites and the enriched pathway analysis, by using KEGG and MetaboAnalyst, provided the first indication that the pathways, such as the glutathione metabolism, is involved in the early metabolic rewiring under the AMP exposure (Figure S6). Thus, we can hypothesize that the AMPs tend to initiate the internalization, oxidative stress, and lipid remodeling but, concurrently, the A549 and THP-1 cells finely tune the metabolism, in order to resist the inflammatory overactivation. This fits into the modern concept about the anti-inflammatory effect of ornithine and its metabolites [97].

On the contrary, the SiO_2_ particles triggered the down-regulation of nine polar metabolites (histidine, deoxyuridine, glutamate, threonine, glycine, glutamine, 2-methylbutyroylcarnitine, methionine-sulfoxide, and hexanoylcarnitine) and the up-regulation of one (sarcosine) metabolite (Figure S5). As previously reported, the SiO_2_ particles increase the aerobic glycolysis and affect the mitochondrial respiration, which reduces the activity of complex I and enhances the activity of complex II, to sustain the cell viability [98]. These mitochondrial alterations are associated with the reductions of the tricarboxylic acid (TCA) cycle intermediates, including glutamate and glutamine, as observed in this study. Moreover, the pathway enrichment analysis disclosed that the main metabolic pathway involved in the response to the SiO_2_ particles was the glycine, serine, and threonine metabolism (Figure S6). It is not a surprising discovery, since the metabolites within this pathway fuel the inflammatory activation of macrophages [99].

By analyzing the levels of cytokines/chemokines (interleukin-6, interleukin-8, and monocyte chemoattractant protein-1; MCP-1) released by the A549/THP-1 cells upon the AMP exposure, the aim was to scrutinize whether the AMP-cell interactions may trigger the acute inflammation that is detectable at the functional, protein level. The results showed that the AMPs did not enhance the release of the inflammatory cytokines/chemokines (Figure S7). This is in line with our previous results demonstrating that no evident effect of the condensate/spatter particles formed during the SLM printing of the different alloys (including a high Ni-containing alloy- Hastelloy X, a maraging steel (18Ni300), an austenitic stainless steel (316 L), and a titanium alloy (Ti6Al4V)) on the release of cytokines/chemokines in THP-1 cells was observed after 24 h of exposure, either on the unprimed or LPS-primed cells [6]. On contrary, the SiO_2_ particles enhanced the release of the cytokines/chemokines and confirmed the results observed at the metabolite level (Figure S6). We can summarize that the different effects of the AMPs and the SiO_2_ particles observed in the Cell Painting assay, lipidomics, metabolomics, and cytokine/chemokine levels clearly disclose their distinct MoA(s).

The integration of the Cell Painting data with the large-scale systems toxicology data, such as those derived from the metabolomics, is sensible to create a sensitive test platform for the detection of the (nano)particle exposure effects. This may have significant implications in situations where low dose (nano)particle exposures may indicate a lack of effects when conventional toxicological screening tools are used.

## 3. Conclusions

Following almost two decades of research, mainly focusing on engineered (nano)particles, it is time to shift our attention to also include the unintentionally formed (nano)particles in various occupational settings and elaborate the tools for safety screening. Adopting NAMs, including in vitro high-throughput and HCS methods, is highly needed, in order to improve the current (nano)particle safety assessment practices. This will promote the development and implementation of alternative methods/strategies to reduce, refine, and replace (3R) in vivo testing, as well as to provide the equivalent or improved scientific quality results more relevant for assessing the (nano)particle safety risks [9].

Following the AMPs characterization, the use of straightforward plate-based bioassays revealed that the AMPs, collected from the filter cartridges of a L-PBF AM machine, induced oxidative stress, most probably, following their internalization. The HCS, by means of the Cell Painting assay, enabled the identification of the cell morphological phenotypes induced by the exposure to the AMPs with the single-cell resolution, which disclosed the effect of the AMPs on the mitochondria, cytoskeleton, and lipid-rich membranes. These findings were further corroborated by the discovery of the impact of AMPs on the membrane remodeling, detected by lipidomics. To improve the understanding of the AMP interactions with the cells from a mechanistic perspective, we profiled the inflammation-related metabolites and found that the cells tended to finely tune their response and did not initiate an inflammatory overactivation. This was also confirmed by the inability of the AMPs to trigger the cytokine/chemokine release, in contrast to the SiO_2_ particles. In short, oxidative stress seems to be a main outcome of the AMP exposure, further orchestrating the complex cellular and molecular events, including the activation of the mitochondria, cytoskeleton disruption, and antioxidant metabolism. The unanswered question is however, for how long can the cells resist before being damaged. To answer this question, we aim, in future studies, to investigate the temporal domain of the cell responses, when repeatedly exposed for prolonged periods to the AMPs. Another question that needs to be answered is whether we can come even closer to understanding of the specific MoAs. We envision to approach this question by matching the morphological profiles obtained in this study with the reference profiles from the high-quality shared databases and repositories.

In conclusion, our study discloses three important aspects of relevance for the nanosafety assessment and for the improved understanding of the toxic effects induced by the inhalation of the (nano)particles, in this study, exemplified for the (nano)particles unintentionally formed in metal additive manufacturing (AMPs):

- A novel method for the nanosafety studies is described and employed as capable of detecting the early changes in the cell morphological phenotypes at low (nano)particle concentrations and able to suggest the prevailing adverse MoAs induced by the (nano)particle-cell interactions. This indicates that the cell stress conditions may be detected upon exposure to the (nano)particles before it can be observed in the reduced cell viability;
- The initial integration of the techniques provides important knowledge for the morphological, lipidomic, and metabolomic signatures as biomarkers of the AMP exposure;
- A proof-of-concept is presented that suggests that the MoAs of the AMPs are complex and, especially at the molecular level, do not always follow a concentration-dependent pattern. We envision future studies to comprehensively elucidate the AMP-cell interactions and MoA in human cells, and to apply lung/bronchial epithelial cells and macrophages as cell models in the Cell Painting profiling.

## 4. Materials and Methods

### 4.1. AMPs and the Characterization Methods

#### 4.1.1. Source, Stock Dispersions, and Endotoxin Test

The AMPs investigated in this study were extracted from filter cartridges used for 2000 process hours in an EOSM290 Laser Powder Bed Fusion (L-PBF) AM machine (EOS GmBH). In brief, the machine used an AM hot work alloy (0.5 wt.% Mn; 5.0 wt.% Cr; 91.4 wt.% Fe; 2.3 wt.% Mo; 0.2 wt.% Si; 0.6 wt.% V) and AM alloy for plastic molding (0.3 wt.% Mn; 12 wt.% Cr; 75.2 wt.% Fe; 1.4 wt.% Mo; 9.2 wt.% Ni; 1.6 wt.% Al; 0.3 wt.% Si). Whilst running the printing process, the laser melting was performed under an argon flow (99.999% purity). The gas entered the printing chamber through an inlet nozzle, and progressively passed over the build plate, collecting the process by-products i.e., smoke, feedstock particles and micron, and nano-sized condensate particles from the process, through the outlet nozzle and onto the filters. The gas was cleaned using a two-stage filter system. The AMPs were extracted from the first filter stage consisting of four cylindrical filter cartridges by gently scraping and without touching the filter surface.

To prepare a stock dispersion, the collected AMP powder was weighed and dispersed in Milli-Q water (18.2 MΩ cm resistivity) to a final concentration of 10 mg/mL, followed by sonication for 20 min using a water bath sonicator Diagenode Bioruptor (Diagenode, Denville, NJ, USA), before dilution in a cell culture medium to the indicated concentrations. Fresh stock dispersions were prepared before each experiment.

The Pierce Chromogenic Endotoxin Quant Kit (Thermo Fisher Scientific, Rockford, IL, USA) was used to detect the presence of endotoxins (bacterial lipopolysaccharide, LPS) in the AMP dispersions, following the manufacturer’s instructions. In brief, 50 µL of the standards, blank (endotoxin-free water), and the AMP dispersions (25, 50, 100 µg/mL, and supernatant of the stock dispersion) were added in the microplates. fifty µL of reconstituted limulus amoebocyte lysate (LAL) reagent was added to each well. The microplates were incubated for 25 min at 37 °C by using a plate heater. Then, 100 µL of pre-warmed chromogenic substrate was added and the plates were incubated for an additional 6 min at 37 °C. Then, 50 µL of 25% acetic acid was added to stop the reaction, and the absorbance was immediately read at 405 nm by using a multi-mode microplate reader FLUOstar Omega (BMG LABTECH, Ortenberg, Germany). The endotoxin contamination of the AMPs was expressed as the endotoxin units (EUs) per mL of the AMP dispersion.

#### 4.1.2. Scanning Electron Microscopy Combined with Energy Dispersive Spectroscopy (SEM-EDS)

The AMPs were characterized by means of SEM using a Zeiss Sigma 300 VP FEG-SEM instrument (Zeiss, Oberkochen, Germany) operated at 20 kV. Prior to the material analysis by SEM, the AMPs were mounted on a carbon adhesive to ensure a good signal to noise ratio. In the analysis, focusing on the size and shape of the particles, the secondary electron detector (SE2-detector) was used. The bulk elemental composition (wt.%) was determined by means of EDS using a TM-1000 tabletop microscope (Hitachi, Japan), using SwiftED-TM software, operated at 15 kV, and Zeiss Sigma 300 VP FEG-SEM, using EDAX TEAM EDS software, operated at 20 kV.

#### 4.1.3. Transmission Electron Microscopy (TEM)

Five µL of the AMP dispersion in Milli-Q water (25 µg/mL) containing 0.1% trehalose were added onto the glow discharge activated (30 s, 1 kV, 100 mA) [100] carbon/formvar coated 300 mesh copper grids for 30 s. The excess liquid was then blotted with a filter paper before the grids were air-dried at room temperature. The analysis was performed using the AnalySis 5.2 software suite (version 5.2; EMSIS, Germany) on images obtained by a Philips CM100 TEM (Philips EO, The Netherlands) instrument equipped with Veleta slow-scan CCD camera (EMSIS, Münster, Germany), at a magnification of 64,000×, giving a pixel size of 0.3 nm. All measurements were conducted at 80 kV. The statistics and size distribution graphical output were carried out in R (R Core Team).

#### 4.1.4. X-ray Photoelectron Spectroscopy (XPS)

The compositional analysis (information depth approx. 5–10 nm) of the outermost surface of the AMPs attached onto the carbon tape was performed by means of XPS (UltraDLD spectrometer, Kratos Analytical, Manchester, UK) using a monochromatic Al X-ray source (10 mA, 15 kV, 150 W). The information was obtained from the attached AMPs covering two different surface areas, each approximately sized 0.35 mm^2^. The wide spectra and the high-resolution spectra (20 eV pass energy) were acquired for Cr 2p, Mn 2p, Fe 2p, Ni 2p, Al 2p, Si 2p, V 2p, O 1s, and C 1s. The binding energy correction was made using the C 1s contamination peak (C-C, C-H) at 285.0 eV. The results are presented as the average relative mass content (wt.%) of the oxidized metals present (Meox/(Meox(tot)) within the outermost surface of the micron- and nano-sized AMPs. Depending on the nanoparticle size, the result may also reflect their bulk content.

#### 4.1.5. Analysis of the Metal Release in the Cell Medium by Atomic Absorption Spectroscopy (AAS)

Prior to the in vitro exposure testing, all experimental vessels and equipment were acid-cleaned with 10% HNO_3,_ for at least 24 h, followed by the four-time rinsing in ultrapure water (resistivity of 18.2 MΩ cm) before being dried in ambient laboratory air. The triplicate samples of the AMPs with one blank sample (without particles) were in parallel exposed to the cell medium (DMEM/nutrient mixture F-12 supplemented with 7.5% FBS) at 37 ± 1 °C for 24 h in dark conditions in an incubator (Edmund Bühler GmbH TH30, Germany), using gentle bilinear agitation (inclination 12°, 22 cycles/min). A mass to solution volume ratio (loading) of 0.1 g/L AMPs was obtained by mixing 1 ± 0.1 mg dry powder with 10 mL cell medium in acid-cleaned vessels. This loading is recommended by the OECD transformation/dissolution protocol for acute aquatic tests [101] and has in previous investigations [102,103,104] been shown to be sufficiently low to avoid the extensive particle wear and agglomeration in the solution. The pH of the cell medium was measured before and after exposure. Following the exposure, 8 mL of the upper portion of the solution was transferred to a new tube and centrifuged (3000× *g* for 10 min) to separate the AMPs from the solution. Five mL of the supernatant was thereafter transferred to a storage vessel and acidified to pH < 2, using 65% ultrapure HNO_3_ prior to the solution analysis, to avoid the precipitation of the released metals in the solution. Further experimental details are given elsewhere [105].

The total amounts of the released Ni and Mn from the AMPs into the cell medium (triplicate readings of each sample) were analyzed by means of graphite furnace atomic absorption spectroscopy (GF-AAS) using a PerkinElmer AA800 analyst instrument (PerkinElmer, Waltham, MA, USA). The calibration curves were based on four standard calibration points at 0, 10, 30, and 100 µg/L. The limits of detection (LODs) and the limits of quantification (LOQs) were 1.0 µg/L and 4.0 µg/L for Ni, and 0.2 µg/L and 1.0 µg/L for Mn. The recoveries of the spiked matrix samples were in the 100–130% range of the nominal values. The released amounts of metals expressed as µg/µg are based on the blank-corrected concentrations of the released metals (µg/L) multiplied by the solution volume (L) and divided by the sample weights (µg) of the AMPs.

### 4.2. Mono- and Co-Culture Cell Models

The U-2 OS cells (Sigma-Aldrich) were cultured in Dulbecco’s Minimum Essential Medium F-12 nutrient mixture (DMEM-F12 + GlutaMAX; Gibco; Life Technologies Ltd., Paisley, UK) supplemented with 7.5% (*v/v*) fetal bovine serum (FBS; Sigma), 10 U/mL penicillin, and 10 μg/mL streptomycin (P/S; Gibco; Life Technologies, Grand Island, NY, USA), and 1× non-essential amino acids (NEAA; Gibco; Life Technologies Ltd., Paisley, UK). The cells were kept at 37 °C in a humidified 5% CO_2_ atmosphere, and after reaching 80 to 90% confluency, they were washed twice with phosphate buffered saline (1× PBS) (Gibco, Life Technologies Ltd., Paisley, UK), detached with 0.05% trypsin-EDTA (Gibco), and reseeded in a fresh complete medium in T75 cell culture flasks (Sarstedt, Nümbrecht, Germany).

The human peripheral blood monocyte cell line THP-1 (InvivoGen, San Diego, CA, USA) was cultured in RPMI-1640 (Gibco) supplemented with 10% FBS, 1% P/S, and 1% L-glutamine. The cells were cultured in T75 cell culture flasks and kept at 37 °C with 5% CO_2_, and passaged every 2–3 days. The cell density was maintained between 6 × 10^5^–1.5 × 10^6^ cells/mL.

The human type II alveolar epithelial cell line A549 from the American Type Culture Collection (ATCC) was cultured in RPMI-1640 (Gibco), supplemented with 10% FBS, 1% P/S, and 1% L-glutamine, in T75 cell culture flasks, and maintained at 37 °C with 5% CO_2_. Once reaching an 80 to 90% confluency, the cells were detached with 0.05% trypsin-EDTA (Gibco) and transferred into fresh R10 medium in T75 flasks.

The co-cultures of the A549/THP-1 cells were established as follows: on day 0, the THP-1 monocytes were seeded at a density of 5 × 10^5^ cells in 24-well plates (Sarstedt). The differentiation into macrophages was carried out with 100 nM phorbol 12-myristate 13-acetate (PMA; Sigma) in a RPMI-1640 medium for 72 h. On day 3, macrophages were washed three times in a RPMI-1640 medium and allowed to rest in fresh medium for an additional 48 h. On day 4, the A549 cells were seeded in 24-well plates at a density of 1.5 × 10^5^ cells/well in 1 mL of medium. On day 5, the macrophages were washed twice with 1× PBS, dissociated with 0.05% trypsin-EDTA, quantified and resuspended in RPMI-1640 medium at the concentration of 6 × 10^5^ cells/mL, and subsequently added on top of the A549 cells in a final volume of 1 mL. Considering that the A549 cells have a doubling time of approximately 22–25 h, the number of THP-1 cells to be seeded was adjusted, in order to achieve a final ratio of 10:1 (A549:THP-1). The co-cultures were left for another 24 h at 37 °C and 5% CO_2,_ prior to the exposure to the AMPs.

### 4.3. Cell Viability and Reactive Oxygen Species (ROS) Detection Assays

To determine the impact of the AMP exposure, the cell viability assays, and ROS detection assay measurements were performed prior to the Cell Painting experiments. For both assays, the U-2 OS cells were seeded at a density of 5000 cells/well using CellCarrier Ultra 96-well black-walled microplates with optically clear flat-bottoms (PerkinElmer) in a final volume of 100 µL. For the A549/THP-1 co-cultures, the same seeding approach was used, as described in the Section 4.2. The cells were incubated for 24 h followed by exposure to a range of AMP concentrations (0, 5, 25, 50, and 100 µg/mL) for an additional 24 h at 37 °C in a humidified incubator under 5% CO_2_.

A HCS LIVE/DEAD Green Kit (Invitrogen; Thermo Fisher Scientific, Eugene, OR, USA) was used to determine the impact of the AMP exposure on the cell viability. Following the exposure, 50 µL of the Image-iT DEAD Green viability stain (prepared in the cell medium) was added to each well, and the cells were incubated for 30 min at 37 °C. The stain is impermeant to healthy cells that can gain entrance when the plasma membrane integrity is compromised. Later, the complete medium was removed, and the cells were fixed with 4% paraformaldehyde (PFA; Thermo Fisher Scientific, Rockford, IL, USA) in PBS, containing Hoechst 33342 (Thermo Scientific) for the cell nuclei labeling and total cell demarcation. Following 15 min of fixation, the staining solution was removed from the plate and the cells were washed three times with 1× PBS. The plates were imaged immediately after labeling, by using a high-throughput imaging platform InCell 2200 HTS (GE Healthcare, NJ, USA).

The Alamar blue assay was used to determine the metabolic activity of the cells as a measure of the cell viability. Following exposure to the AMPs, the cells were washed twice with pre-warmed 1× PBS, and 10% alamarBlue™ HS Cell Viability Reagent (Invitrogen; Thermo Fisher Scientific, Eugene, OR, USA) in DMEM-F12 + GlutaMAX was added, and the cells were incubated for 3 h at 37 °C. Then, the fluorescence was read using the FLUOstar Omega microplate reader at 540/590 nm excitation and emission. The interference control samples (10% Alamar blue in medium + AMPs) and the blank control samples (10% Alamar blue in medium without cells) were included.

The ROS detection was performed using the CellROX Oxidative Deep Red Reagent (Invitrogen; Thermo Fisher Scientific, Eugene, OR, USA), as per the instructions of the manufacturer. Following the AMP exposure, 100 µL of the CellROX Reagent (prepared in the cell medium) was added to the wells, reaching a final concentration of 5 µM and incubated for 30 min at 37 °C. The CellROX Deep Red is a cell-permeable reagent localized in the cytoplasm and exhibits a strong fluorogenic signal in its reduced state. Following the labeling, the medium was removed, and the cells were washed three times with 1× PBS. In addition, the cells were fixed with 4% PFA in 1× PBS, containing Hoechst 33342. The plates were imaged by the high-throughput imaging platform InCell 2200 HTS (GE Healthcare) and the fluorescent signal was quantified by means of the ImageJ software, version 1.8.0 (NIH, USA).

### 4.4. AMPs Internalization Analysis

The control and AMPs-exposed (50 µg/mL) U-2 OS cells adhering to 10 mm circular glass coverslips (Epredia, Braunschweig, Germany) were fixed with 3% EM-graded glutaraldehyde (Sigma-Aldrich) in 1× PBS. Subsequently, the coverslips were washed three times with PBS, postfixed in PBS buffered 1% OsO_4_, extensively washed in PBS, dehydrated through an alcohol series (25, 50, 75, 90, 96, and 100%), and dried in a K850 critical point dryer (Quorum Technologies Ltd., Ashford, UK). Half of each coverslip was sputter-coated with 3 nm of platinum; the other half with 6 nm of carbon using a high-resolution turbo-pumped sputter coater Q150T (Quorum Technologies Ltd., Ashford, UK). SEM in the secondary electrons and EDS were carried out at 5 kV in FEI Nova Nano SEM 450 (FEI, Brno, Czech Republic) equipped with an Ametek^®^ EDAX Octane plus SDD detector and TEAM-EDS analysis systems (AMETEK B.V., Tilburg, The Netherlands).

### 4.5. Cell Painting and Data Analysis

#### 4.5.1. Cell Seeding and AMP Exposure

For each Cell Painting experiment, a single vial of the passage 3–6 U-2 OS cells was thawed and cultured through four to seven consecutive passages. Using a Thermo Scientific Multidrop Combi dispenser, the cells were seeded at a density of 5000 cells/well in CellCarrier Ultra 96-well black-walled microplates with optically clear flat-bottoms (PerkinElmer) in a volume of 100 µL. The plates were incubated overnight for 24 h at 37 °C at a 5% CO_2_ atmosphere, to allow for the cell attachment. The outer microplate wells were filled with 200 µL of 1× PBS and were excluded from the experiment to avoid edge effects. Following 24 h, 100 µL of warm cell medium containing 2× AMPs concentrations was added to the microplate wells containing the cells. The cells were exposed for 24 h to final 1× AMP concentrations set at 0, 0.156, 0.313, 0.625, 1.25, 2.5, 5, 25, 50, and 100 µg/mL.

#### 4.5.2. Cell Staining and Image Acquisition

Following the exposure, the Cell Painting followed the protocol described by Bray et al. [11]. Briefly, the microplates were inverted, and the cell medium was discarded. In order to precisely and accurately dispense the exact same volume of fluorescent dye, we used an electronic multi-dispensing micropipette (Integra). In the next step, 30 µL of MitoTracker (Invitrogen; Thermo Fisher Scientific, Eugene, OR, USA) in a pre-warmed culture medium (DMEM-F12 + GlutaMAX) was added and the cells were incubated for 30 min at 37 °C at a 5% CO_2_ atmosphere. Following the incubation, 10 µL of 16% paraformaldehyde (PFA) was added, reaching a final concentration of 4% PFA. The cells were fixed for 20 min in the dark at room temperature. Then, the MitoTracker and PFA solutions were removed, and the cells were washed two times (70 μL, 1× PBS) by a Thermo Scientific Multidrop Combi set in washing mode. A cell-staining cocktail was prepared by adding Hoechst 33342 (Thermo Fisher Scientific), SYTO 14 green (Invitrogen), concanavalin A/Alexa Fluor 488 (Invitrogen), wheat germ agglutinin/Alexa Fluor 555 (Invitrogen), phalloidin/Alexa Fluor 568 (Invitrogen), and 0.1% Triton X-100 in 0.1% bovine serum albumin (BSA), prepared in 1× PBS. The cells were stained in a final volume of 30 µL for 30 min at room temperature and protected from the light. Then, the microplates were washed four times (70 μL, 1× PBS). PBS used for washing and the preparation of the stain solutions was always pre-equilibrated to room temperature.

Following the washing, the cells were left in 70 μL 1× PBS and imaged on a high-throughput imaging platform (InCell 2200 HTS system), using a 20× objective. The z-offsets for each channel were optimized by examining the randomly selected wells/fields across the microplates with the goal of acquiring sharp, “in focus” images for the cell compartments of interest. In brief, nine images corresponding to nine different fields of view in each well were captured in five different fluorescence channels to capture the DNA (Hoechst), mitochondria (MitoTracker), Golgi apparatus, and plasma membrane (wheat germ agglutinin), F-actin (phalloidin), nucleoli and RNA (SYTO 14), and the endoplasmic reticulum (concanavalin A/Alexa Fluor 488). The excitation spectra were set to 390/18 nm (Hoechst), 632/22 nm (MitoTracker), 575/25 nm (phalloidin and wheat germ agglutinin), 542/27 nm (SYTO 14) and 475/28 nm (concanavalin A). The emission filters were set to detect the signals between 435/48 nm (Hoechst), 679/34 nm (MitoTracker), 620/30 nm (wheat germ agglutinin and phalloidin), 597/45 nm (SYTO 14), and 511/25 (concanavalin A).

#### 4.5.3. Image Processing and Cell Profiling

In order to extract the morphological features, the obtained images were analyzed using the open-source image analysis software CellProfiler v. 4.2.1 (www.cellprofiler.org (accessed on 24 March 2022)) [106]. CellProfiler was running the illumination correction (JUMP_illum_LoadData_v1.cppipe) and analytical (JUMP_analysis_v3.cppipe) pipelines developed by the Broad Institute for the JUMP—Cell Painting Consortium (https://github.com/broadinstitute/imaging-platform-pipelines/tree/master/JUMP_production (accessed on 24 March 2022)). We used the pipeline to segment the cells, distinguish between the nuclei and cytoplasm, and then measured the specific features related to the channels captured, e.g., fluorescence intensity, texture, granularity, density, location, and various other measurements for each single cell. Following the analysis pipeline, we obtained 3676 feature measurements for each of 44,000 (in average) cells, per treatment (cell-based morphological profiles). Additionally, we obtained 1001 feature measurements, based on the averaged data from each image (approximately 200–250 cells per image; image-based morphological profiles).

Following the extraction of morphological single-cell features by CellProfiler, we utilized the quality control, feature selection, and normalization procedures. First, we removed the data collected from the wells with fewer than 500 cells, and from the images (microscope fields of view) that had fewer than 30 cells. The cells that had missing values in their profiles and block-listed features [107] were excluded from further analysis. The normalization against the unexposed controls was employed for the single-cell profiles [11]. Each feature value from a single-cell profile was subtracted by the median value of the unexposed control and after that divided by the median of the absolute deviation value of the control. Such an unexposed control normalization step enabled a possibility to compare the morphological profiles of the AMP-exposed cells. Then to correct the impact of the various technical biases through the plates (“batch effect”), we employed the pyComBat version 0.3.1 [108] software package that is a Python implementation of ComBat [109]. The well-level value profiles were obtained by the median calculations of the normalized cell-level values. The treatment-level values were calculated as the mean values of the well-level values from all wells in one microplate treated with the same AMP concentration. The averaged treatment-level values obtained from the three microplates (three biological replicates) were used for the graphical heatmap presentations. The self-developed Python scripts, available upon request, were used for all the above-described steps.

#### 4.5.4. Univariate, Unsupervised, and Supervised Multivariate Analyses

The Cell Painting features were analyzed using univariate, unsupervised multivariate, and supervised multivariate approaches. The ANCOVA models were implemented for each Cell Painting feature (image level) by modelling the feature values as a function of the concentration of the AMPs (as covariate) and the plate-batch effect (3-level factor). The resulting *p*-values testing the significance of a non-zero slope for the feature response as dependent on the AMP concentration were collected and corrected for multiplicity taking a Bonferroni correction approach for a significance level of 5% (and a family of 977 tests).

For the image level morphological profiles, the “batch effect” was corrected through the plates by using the ComBat software package implemented in R [109]. To visualize the high-dimensional data in the lower dimensional space, we have applied the uniform manifold approximation and projection (UMAP) [78] and all 1620 observations were displayed as embedded in a 2-dimensional UMAP space. The AMPs concentration levels were color-coded for the display. We employed 200 neighbors for the UMAP dimensionality reduction.

Finally, sPLS-DA was employed to analyze the potential of the Cell Painting feature to predict the AMP concentrations in the new samples. Hereto, we used the approach by Chung and Keles [110], as implemented in R [111] (R package version 2.2–3) To assess the expected performance of the predictions of the high vs. low AMP concentrations from the Cell Painting data, a systematic cross-validation approach was implemented. For each of the three runs, the data for one of the three plates was omitted before training the sPLS-DA model. The concentrations for the left-out data were then predicted, based on their Cell Painting data and the fitted model. The results for the prediction performance were averaged between the three runs. The selected variables for the three runs were collected and the numbers were reported together with the number of the variables selected by all three runs.

### 4.6. Multiplex Immunoassay

Prior to the AMP exposure, the medium from the A549/THP-1 co-cultures was removed. Then, the cells were exposed to the AMPs dispersed to 5, 25, 50, and 100 µg/mL nominal concentrations in 500 µL of the RPMI-1640 medium. The SiO_2_ particles (Invivogen) of a final concentration of 70 µg/mL were used as the positive control. Following 24 h of exposure, 300 µL of the cell culture supernatant was collected and centrifuged at 5000× *g* for 5 min, aliquoted and stored at −80 °C until the analysis. The levels of the released inflammatory cytokines/chemokines were assessed using the immunoassay LEGENDplex Human Inflammation Panel from BioLegend (San Diego, CA, USA), according to manufacturer’s instructions, using the flow cytometer Accuri C6 (Becton Dickinson, San Jose, CA, USA). The exposure experiments were performed three times with two technical replicates for each condition.

### 4.7. Lipidomic Analysis

Following the collection of the cell culture supernatants (see section Multiplex Immunoassay), the adherent A549/THP-1 co-cultures were gently detached using a cell scraper and transferred, along with the remaining supernatant, to the microcentrifuge tubes. The cells were centrifuged for 10 min at 10,000 g at 4 °C. Following the centrifugation, the supernatants were aspirated and discarded, and the cell pellets were stored at −80 °C. For the lipidomic analysis, the cell pellets were randomized and analyzed, as described below. Then, 150 µL of 0.9% NaCl was added to the cell pellets. Following the sonication, 20 μL of the cell homogenate was extracted with 150 µL of CHCl_3_: MeOH (2:1, *v/v*), containing 2.5 µg/mL of the internal standard solution (1,2-diheptadecanoyl-sn-glycero-3-phosphoethanolamine (PE(17:0/17:0)), N-heptadecanoyl-D-erythro-sphingosylphosphorylcholine (SM(d18:1/17:0)), N-heptadecanoyl-D-erythro-sphingosine (Cer(d18:1/17:0)), 1,2-diheptadecanoyl-sn-glycero-3-phosphocholine (PC(17:0/17:0)), 1-heptadecanoyl-2-hydroxy-sn-glycero-3-phosphocholine (LPC(17:0)) and 1-palmitoyl-d31-2-oleoyl-sn-glycero-3-phosphocholine (PC(16:0/d31/18:1)) and, triheptadecanoylglycerol (TG(17:0/17:0/17:0)). The samples were vortexed and left to stand on ice for 30 min before centrifugation (9400× *g*, 3 min). Then, 60 µL of the lower layer of was collected and diluted with 60 µL of CHCl_3_: MeOH. The samples were kept at −80 °C, until analysis.

The samples were analyzed using an ultra-high-performance liquid chromatography quadrupole time-of-flight mass spectrometry (UHPLC-Q-TOF-MS from Agilent Technologies (Santa Clara, CA, USA). The analysis was carried out on an ACQUITY UPLC^®^ BEH C18 column (2.1 mm × 100 mm, particle size 1.7 μm) by Waters (Milford, MA, USA). The quality control was performed throughout the dataset by including blanks, pure standard samples, extracted standard samples, and control plasma samples. The eluent system consisted of (A) 10 mM NH4Ac in H_2_O and 0.1% formic acid and (B) 10 mM NH4Ac in ACN: IPA (1:1) and 0.1% formic acid. The gradient was as follows: 0–2 min, 35% solvent B; 2–7 min, 80% solvent B; 7–14 min 100% solvent B. The flow rate was 0.4 mL/min. The mass spectrometry data processing was performed using the open-source software package MZmine 2.51 [112]. The identification of the lipids was achieved using a custom database search and the data were normalized using internal standards, followed by the calculation of the concentrations, based on the lipid-class concentration curves. An aliquot of each sample was collected and pooled, and used as a quality control sample, together with the NIST SRM1950 reference plasma sample (an in-house pooled serum sample).

The calibration curves (100–2500 ng/mL) using 1-hexadecyl-2-(9Z-octadecenoyl)-sn-glycero-3-phosphocholine (PC(16:0e/18:1(9Z))), 1-(1Z-octadecenyl)-2-(9Z-octadecenoyl)-sn-glycero-3-phosphocholine (PC(18:0p/18:1(9Z))), 1-stearoyl-2-hydroxy-sn-glycero-3-phosphocholine (LPC(18:0)), 1-oleoyl-2-hydroxy-sn-glycero-3-phosphocholine (LPC(18:1)), 1-palmitoyl-2-oleoyl-sn-glycero-3-phosphoethanolamine (PE(16:0/18:1)), 1-(1Z-octadecenyl)-2-docosahexaenoyl-sn-glycero-3-phosphocholine (PC(18:0p/22:6)), and 1-stearoyl-2-linoleoyl-sn-glycerol (DG(18:0/18:2)), 1-(9Z-octadecenoyl)-sn-glycero-3-phosphoethanolamine (LPE(18:1)), N-(9Z-octadecenoyl)-sphinganine (Cer(d18:0/18:1(9Z))), 1-hexadecyl-2-(9Z-octadecenoyl)-sn-glycero-3-phosphoethanolamine (PE(16:0/18:1)) from Avanti Polar Lipids, 1-Palmitoyl-2-Hydroxy-sn-Glycero-3-Phosphatidylcholine (LPC(16:0)), 1,2,3 trihexadecanoalglycerol (TG(16:0/16:0/16:0)), 1,2,3-trioctadecanoylglycerol (TG(18:0/18:0/18:)), and 3β-hydroxy-5-cholestene-3-stearate (ChoE(18:0)), 3β-Hydroxy-5-cholestene-3-linoleate (ChoE(18:2)) from Larodan, were prepared to in CHCl_3_:MeOH, 2:1, *v/v*), including 1250 ng/mL of each internal standard.

### 4.8. Metabolomic Analysis

Briefly, the pellets of the A549/THP-1 co-cultures were dissolved in 150 µL sodium chloride solution (0.9% in water). Then, 80 µL of the homogenized samples was transferred to the microcentrifuge tubes and 180 µL of the internal standard (IS) solution was added. The IS solution was prepared by filling up 10 µL of the IS (D5-Glutamic acid, D4-3-Hydroxybutyric acid, D5-DL-1AA, D8-Valine, D3-DL-Ferulic acid, D4-Succinic acid, Heptadecanoic acid, D2-Propionic acid, D3-L-Carnitine methyl, D8-Butyric acid) to 10 mL with methanol. The samples were vortexed, ultrasonicated for 3 min, and then centrifuged for 5 min at 10,000 rpm. Then, 180 µL of the supernatant was transferred to LC-vials and evaporated to dryness. The samples were resuspended with 40 µL methanol and water (1:1). The ultra-high pressure liquid chromatography-tandem mass spectrometry measurements were performed on an Acquity UHPLC coupled to a Xevo TQ-S triple quadrupole mass spectrometer (MSMS) (Waters Corporation, Milford, MA, USA). Two µL of the sample extract was injected onto a 1.7 µm, 2.1 mm × 150 mm Acquity BEH AMIDE HILIC column, in combination with a guard column. The column temperature was 30 °C. The gradient mobile phases were, A (20 mM ammonium formate in 100% Milli-Q and 0.1% formic acid) and B (0.1% formic acid in 100% acetonitrile). The flow rate was 0.3 mL/min and the total run time per sample was 20 min. The analysis was performed using electrospray ionization, operating in the positive ion mode. The source temperature was 150 °C and the desolvation temperature was 350 °C. The cone gas flow was 150 L/h and the desolvation gas flow was 700 L/h. The multiple reaction monitoring (MRM) acquisition mode was selected for the relative quantification of the metabolites with an individual span time of 0.1 s, given in their individual MRM channels.

Overall, the method used to determine the polar metabolites in all cell samples (N = 36; three biological replicates with two technical repetitions per each exposure scenario) was successfully validated, in terms of the recovery and precision. The internal standard recoveries ranged from 82–101%. The precision of the method was determined using the interspaced injections of an in-house pool plasma QC reference sample (N = 8), whose relative standard deviation average ranged between 11 and 58%. The LOD was defined as the average amount of trace of each analyte in the blank samples plus three standard deviations. The LOQ was defined as the average amount of the trace of each analyte in the blank samples plus five standard deviations.

### 4.9. Statistical Analysis

All experiments were performed at least in triplicates with two technical replicates. The statistical analysis of the cytokine/chemokine levels is presented as the mean ± standard deviation (SD). The statistical differences were assessed by a one-way ANOVA followed by the post hoc Tukey test. The significance was rated as *p* < 0.05. The statistical analysis and graphs (ROS and cytokine/chemokine levels) were performed in GraphPad Prism (version 9.1; GraphPad software Inc., La Jolla, CA, USA). A web server MetaboAnalyst was used for the statistical analyses of the lipidomics and metabolomics data, including hierarchical clustering heatmaps [113]. The multiplicity was adjusted, as specifically indicated, for each analysis step.

## Data Availability

The data presented in this study are available upon reasonable request from the corresponding author.

## Supplementary Materials

The following supporting information can be downloaded at: https://www.mdpi.com/article/10.3390/cells12020281/s1, Figure S1: SEM-EDS mapping of C, Fe, Cr, Mn, Mo, Al, Ni, Si, V, O, and S reflecting the AMPs bulk chemical composition; Figure S2: Quantitative summary of the morphological effects for the SiO_2_ particles; Figure S3: Alamar blue viability assay for the A549/THP-1 co-culture exposed to a range of the AMP concentrations; Figure S4: Volcano plot on the down-regulation (blue) of the lipids after 24 h of cell exposure to the 70 μg/mL SiO_2_ particles; Figure S5: Volcano plot on the up- (red) and down-regulation (blue) of the polar metabolites after 24 h cell exposure to the 70 μg/mL SiO_2_ particles; Figure S6: Quantitative pathway enrichment of the differential metabolites obtained by MetaboAnalyst for the AMP-exposed cells (left panel) and for the SiO_2_-exposed cells (right panel); Figure S7: Cytokine/chemokine release in the A549/THP-1 co-culture model exposed to the AMPs, and SiO_2_ particles (positive control) for 24 h; Table S1: Comparative analysis of the relative mass composition of the bulk material and the EDS and XPS analysis of the powder collected from the filter.
