# Supplementary material for: A Novel Nanosafety Approach Using Cell Painting, Metabolomics, and Lipidomics Captures the Cellular and Molecular Phenotypes Induced by the Unintentionally Formed Metal-Based (Nano)Particles
Source: Cells. 2023 Jan 11;12(2):281. doi: 10.3390/cells12020281 (PMC9856453; doi:10.3390/cells12020281)
Supplement: Supplementary file 1 [file cells-12-00281-s001.zip › cells-2114093-supplementary.pdf]

Supplementary Materials

A Novel Nanosafety Approach Using Cell Painting, Metabolomics, and Lipidomics Captures the Cellular and Molecular Phenotypes induced by the Unintentionally Formed Metal-Based (Nano)Particles

Andi Alijagic [1,2,3,*], Nikolai Scherbak [1], Oleksandr Kotlyar [1,4], Patrik Karlsson [5], Xuying Wang [6], Inger Odnevall [6,7,8], Oldřich Benada [9], Ali Amiryousefi [3], Lena Andersson [2,3,10], Alexander Persson [2,3], Jenny Felth [11], Henrik Andersson [11], Maria Larsson [1], Alexander Hedbrant [2,3], Samira Salihovic [1,2,3], Tuulia Hyötyläinen [1], Dirk Repsilber [3], Eva Särndahl [2,3] and Magnus Engwall [1]

[1] Man-Technology-Environment Research Center (MTM), Örebro University, SE-701 82 Örebro, Sweden
[2] Inflammatory Response and Infection Susceptibility Centre (iRiSC), Faculty of Medicine and Health, Örebro University, SE-701 82 Örebro, Sweden
[3] Faculty of Medicine and Health, School of Medical Sciences, Örebro University, SE-701 82 Örebro, Sweden
[4] Centre for Applied Autonomous Sensor Systems (AASS), Mobile Robotics and Olfaction Lab (MRO), Örebro University, SE-701 82 Örebro, Sweden
[5] Department of Mechanical Engineering, Örebro University, SE-701 82 Örebro, Sweden
[6] KTH Royal Institute of Technology, Department of Chemistry, Division of Surface and Corrosion Science, SE-100 44 Stockholm, Sweden
[7] AIMES—Center for the Advancement of Integrated Medical and Engineering Sciences at Karolinska Institutet and KTH Royal Institute of Technology, SE-100 44 Stockholm, Sweden
[8] Department of Neuroscience, Karolinska Institutet, SE-171 77 Stockholm, Sweden
[9] Institute of Microbiology of the Czech Academy of Sciences, 140 00 Prague, Czech Republic
[10] Department of Occupational and Environmental Medicine, Örebro University Hospital, SE-701 85 Örebro, Sweden
[11] Uddeholms AB, SE-683 85 Hagfors, Sweden
* Correspondence: andi.alijagic@oru.se

Table S1. Comparative analysis of the relative mass composition of bulk material and EDS and XPS analysis of the powder collected from the filter.

	**Relative mass composition**							
**Element**	**Mn**	**Cr**	**Fe**	**Mo**	**Ni**	**Al**	**Si**	**V**
**AM hot work alloy**	0.005	0.050	0.914	0.023			0.002	0.006
**AM alloy for plastic molding**	0.003	0.120	0.752	0.014	0.092	0.016	0.003	
**Average (bulk)**	0.004	0.085	0.833	0.019	0.092	0.016	0.003	0.006
**XPS**	0.090	0.026	0.884		0	0	0	0
	0.087	0.022	0.981		0	0	0	0
**EDS**	0.022	0.053	0.914	0.001		0.003	0.003	0.004
	0.024	0.066	0.897		0.002	0.002	0.007	0.002
	0.023	0.058	0.901		0.011	0.002	0.005	
	0.028	0.072	0.868	0.002	0.019	0.007	0.003	0.001
	0.021	0.065	0.900		0.010	0.001	0.002	0.001
	0.030	0.053	0.909		0.001	0.002	0.005	
	0.028	0.060	0.898	0.003		0.007	0.004	
	0.024	0.066	0.898			0.003	0.008	0.001
**EDS average**	0.025	0.062	0.898	0.002	0.009	0.003	0.005	0.002

`

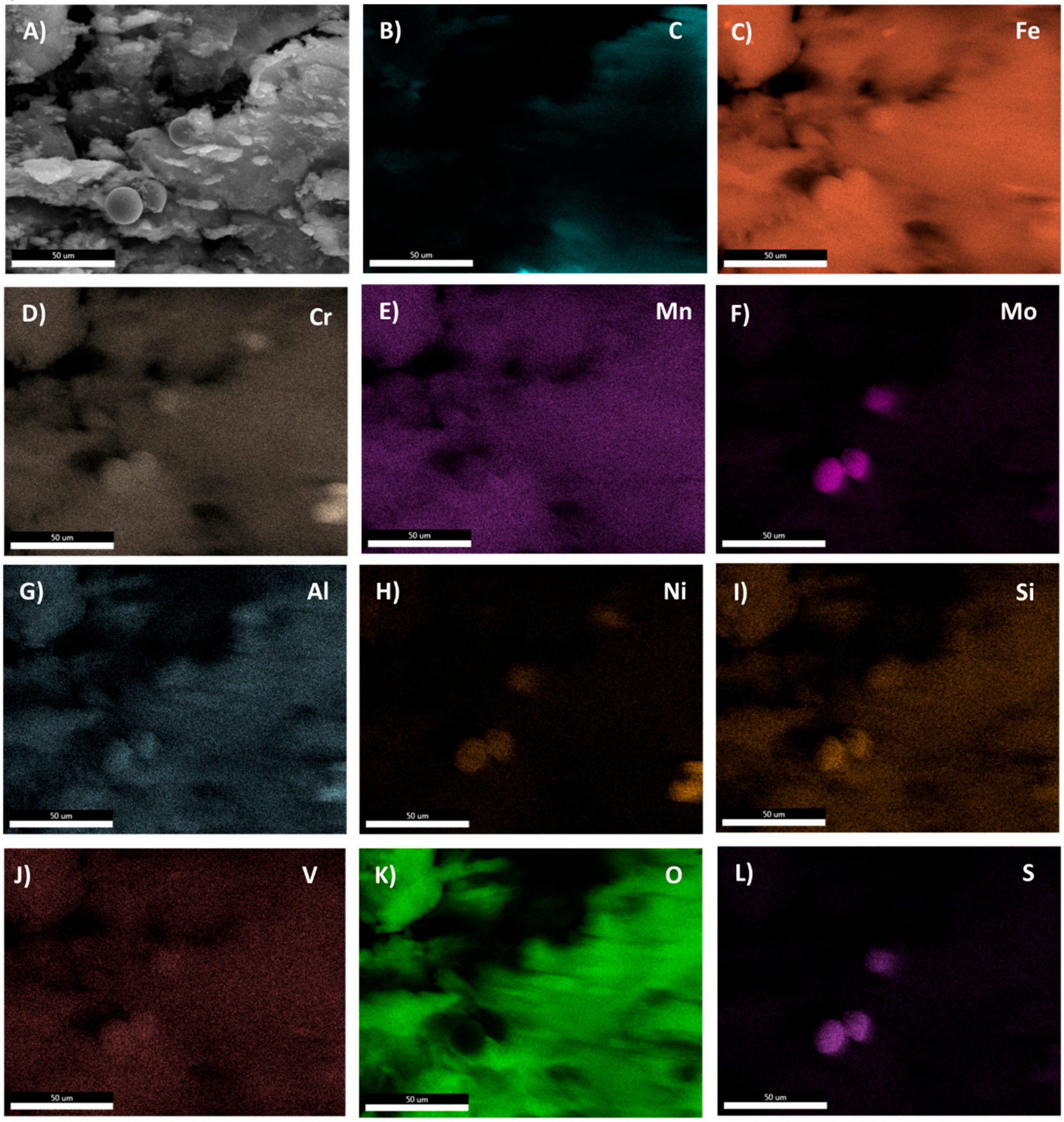

Figure S1. SEM-EDS mapping of C, Fe, Cr, Mn, Mo, Al, Ni, Si, V, O, and S reflecting the AMPs ((nano)particles unintentionally emitted at metal AM occupational settings) bulk chemical composition.

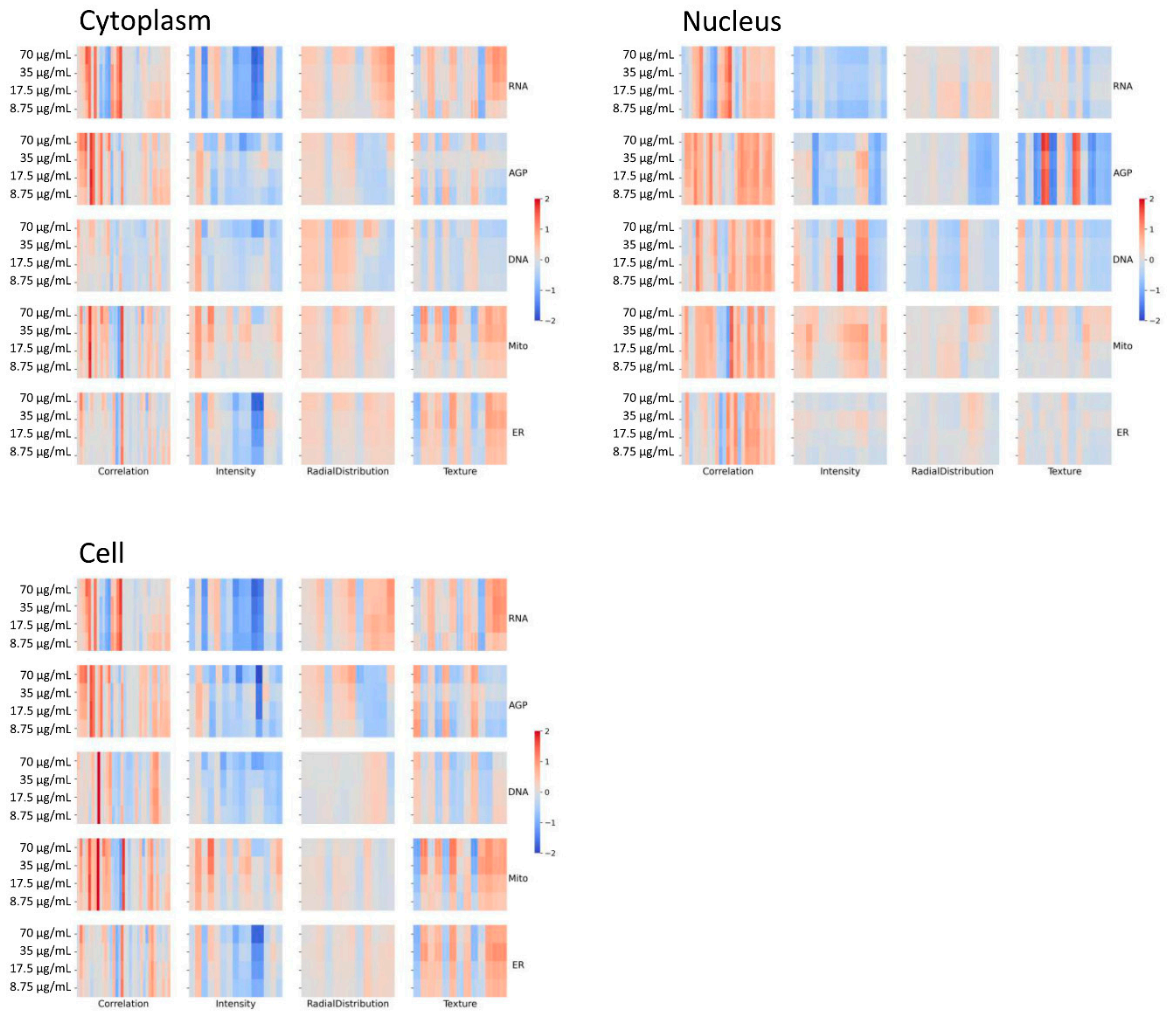

Figure S2. Quantitative summary of morphological effects for the SiO_2 particles. The columns of the heatmap represent individual morphological features, organized by compartment: cytoplasm, nuclei, and cell; and by fluorescent channel: nuclei (DNA), actin cytoskeleton/Golgi/plasma membrane (AGP), endoplasmic reticulum (ER), RNA/nucleoli (RNA), and mitochondria (Mito). The colors represent the fold change in a measured feature with respect to unexposed control cells. The rows correspond to individual concentrations of SiO_2 particles. Exposure concentrations are listed in descending order from top to bottom.

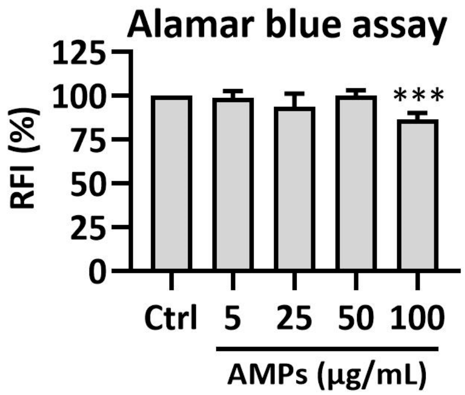

Figure S3. Alamar blue viability assay for A549/THP-1 co-culture exposed to (nano)particles unintentionally emitted at metal AM occupational settings (AMPs). Bar plots represent mean ± SD of three experiments with two technical replicates. ***$P<0.001$. RFI – relative fluorescence units.

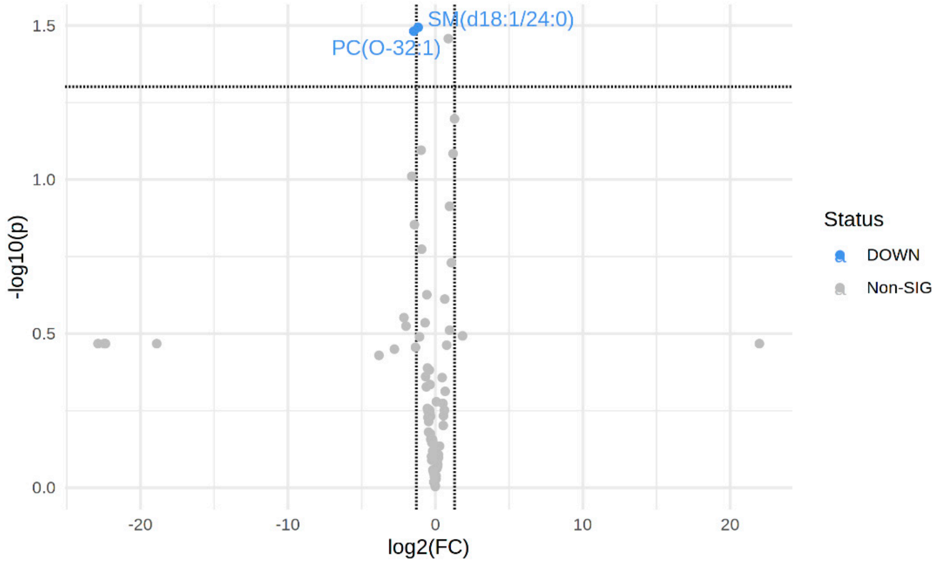

Figure S4. Volcano plot on down-regulation (blue) of lipids after 24 h of cell exposure to 70 µg/mL SiO_2 particles. The log2 (FC – fold change) reflect the relative abundance of lipids in SiO_2-exposed- and control cells. The y-axis represents the −log10 (p-value) between exposed and control samples.

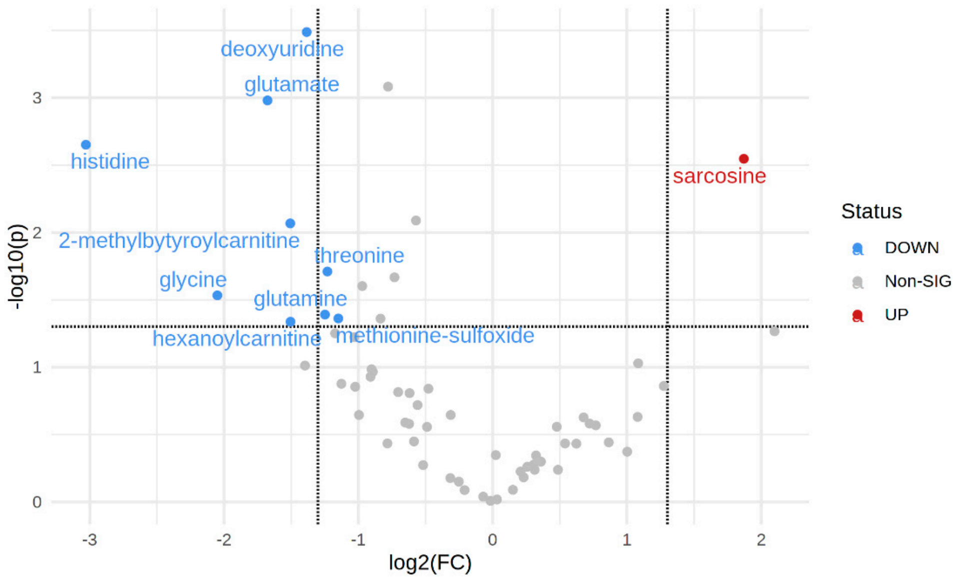

Figure S5. Volcano plot on up- (red) and down-regulation (blue) of polar metabolites after 24 h cell exposure to 70 μg/mL SiO_2 particles. The log2 (FC – fold change) reflect the relative abundance of polar metabolites in SiO_2-exposed- and control cells. The y-axis represents the −log10 (p-value) between exposed and control samples.

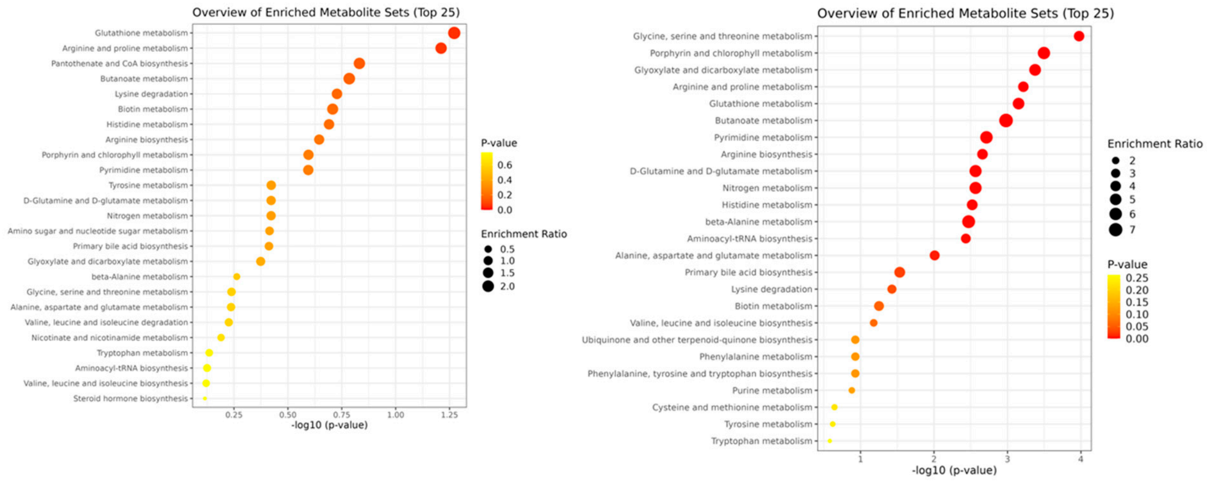

Figure S6. Quantitative pathway enrichment of differential metabolites obtained by MetaboAnalyst for AMP-exposed cells (left panel) and for SiO_2-exposed cells (right panel). AMPs – (nano)particles unintentionally emitted at metal AM occupational settings.

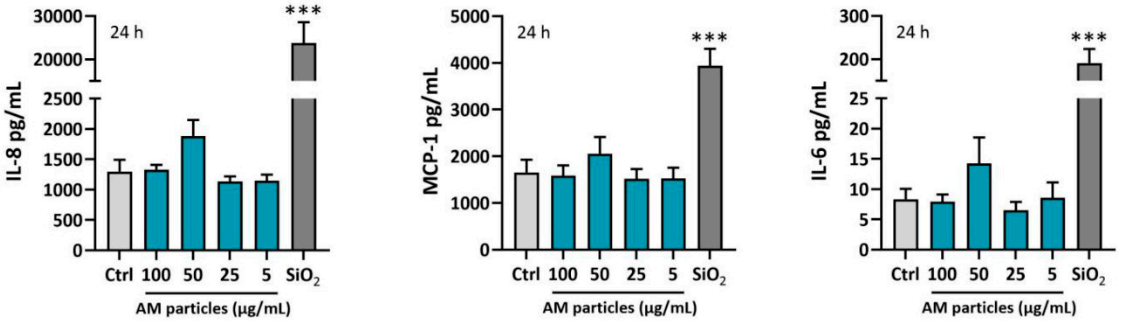

Figure S7. Cytokine/chemokine release in A549/THP-1 co-culture model exposed to (nano)particles unintentionally emitted at metal AM occupational settings (AMPs), and SiO_2 particles (positive control) for 24 h. Data is presented as mean ± SD (n = 3 independent experiments with 2 biological replicates). The asterisks denote statistical significance compared to respective control (***$p < 0.001$). IL-8 – Interleukin-8; IL-6 – Interleukin-6; MCP-1 – Monocyte Chemoattractant Protein-1.
